# The Mammary Gland: Basic Structure and Molecular Signaling during Development

**DOI:** 10.3390/ijms23073883

**Published:** 2022-03-31

**Authors:** Swarajit Kumar Biswas, Saswati Banerjee, Ginger Wendolyn Baker, Chieh-Yin Kuo, Indrajit Chowdhury

**Affiliations:** 1 CSI Laboratories, Inc., Alpharetta, GA 30004, USA; skbiswas135@gmail.com; 2 Department of Physiology, Morehouse School of Medicine, Atlanta, GA 30310, USA; sbanerjee@msm.edu; 3 Department of Obstetrics and Gynecology, Morehouse School of Medicine, Atlanta, GA 30310, USA; gbaker65@gmail.com; 4 Department of Biology, Georgia State University, Atlanta, GA 30302, USA; chiehyin.kuo@gmail.com

**Keywords:** mammary gland, signaling, development

## Abstract

The mammary gland is a compound, branched tubuloalveolar structure and a major characteristic of mammals. The mammary gland has evolved from epidermal apocrine glands, the skin glands as an accessory reproductive organ to support postnatal survival of offspring by producing milk as a source of nutrition. The mammary gland development begins during embryogenesis as a rudimentary structure that grows into an elementary branched ductal tree and is embedded in one end of a larger mammary fat pad at birth. At the onset of ovarian function at puberty, the rudimentary ductal system undergoes dramatic morphogenetic change with ductal elongation and branching. During pregnancy, the alveolar differentiation and tertiary branching are completed, and during lactation, the mature milk-producing glands eventually develop. The early stages of mammary development are hormonal independent, whereas during puberty and pregnancy, mammary gland development is hormonal dependent. We highlight the current understanding of molecular regulators involved during different stages of mammary gland development.

## 1. Introduction

**Figure 1:**
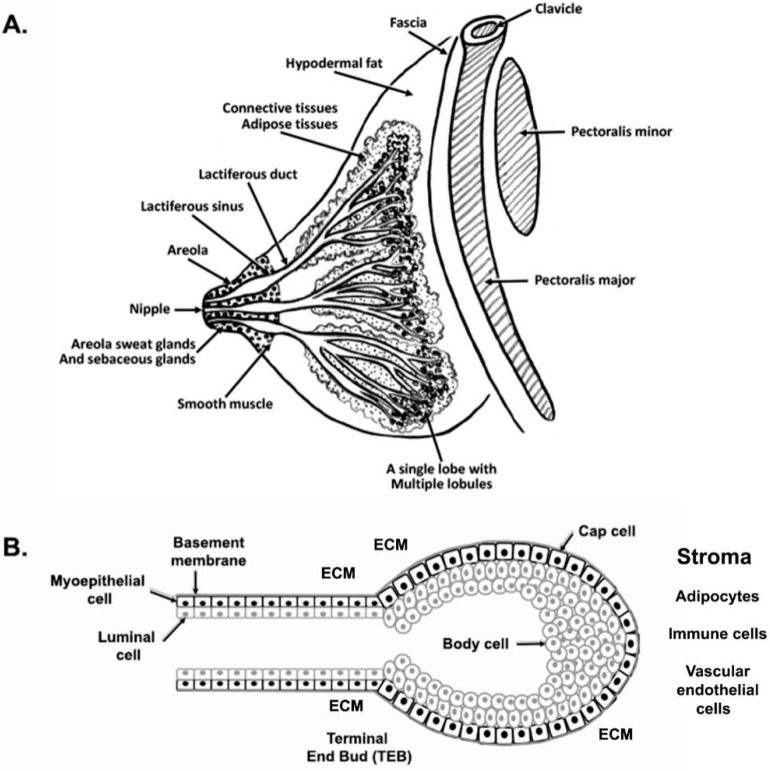
The mammary gland. (**A**) Schematic representation of adult human mammary gland anatomy. The mammary gland (exocrine sudoriferous or sweat gland) is located on the ventral chest wall superficial to the pectoralis muscles. The basic components of a mature mammary gland are the alveoli (hollow cavities, a few millimeters large) lined with milk-secreting cuboidal cells and surrounded by myoepithelial cells. These alveoli join together to form lobules surrounded by adipose and connective tissues. Each lobule has a lactiferous duct that drains into openings in the nipple through lactiferous sinus. The nipple is surrounded by the pigmented areola with sweat and sebaceous glands. (**B**) Schematic representation of a terminal end bud (TEB). Mature ducts are comprised of luminal epithelial cells surrounded by contractile myoepithelial cells, fibrous connective tissue, and variable amounts of adipose tissue. At puberty, these swell into TEBs. TEBs consist of multiple layers of body cells and a single layer of cap cells at the leading edge. The subtending ducts are surrounded by a variety of stromal cells or connective tissues with extracellular matrix (ECM) protein supports the mammary glands. The major components of stromal connective tissues are adipocytes, fibroblasts, vascular endothelial cells, a variety of innate immune cells (both macrophages and mast cells), and nerves. The fatty stroma is the supportive network for the epithelium bi-layered structure and provides nutrients, blood supply, and immune defenses besides the physical structure to the gland. TEBs are regulated hormonally by estrogens (E2) and progesterone (P4) from the corpus luteum and placenta, prolactin (PRL) from the anterior pituitary, and oxytocin from the posterior pituitary gland. TEBs respond to autonomic nervous system impulses.

**Figure 2:**
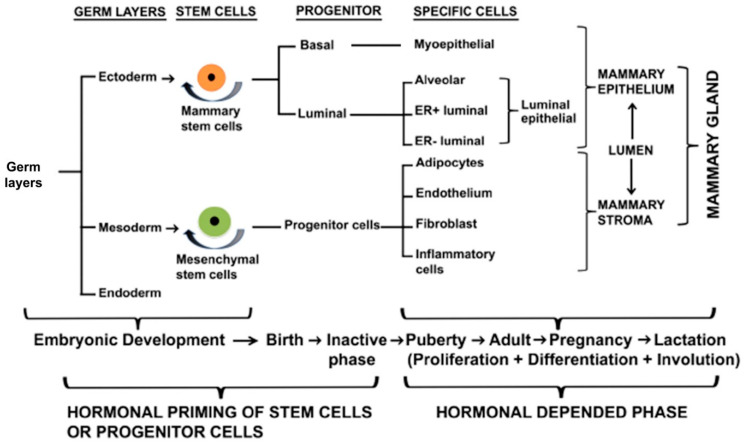
A schematic representation of the origin of epithelial–mesenchymal cells and prenatal mammary development from embryonic to adult stage. The primary mesenchyme forms with gastrulation in mammals provide the first distinction between epithelial and mesenchymal phenotypes. Cells exhibiting a mesenchymal phenotype provide a supportive structure to the epithelial cells by producing the extracellular matrix (ECM). The transition of epithelial and mesenchymal cells forms the primary mesenchyme and three primitive germ layers, namely, ectoderm, mesoderm, and endoderm. The mammary epithelium comprises several cell lineages, including luminal, alveolar, and myoepithelial cells. The mammary epithelium is maintained by the presence of multipotent mammary stem cells (MaSCs). MaSCs provide unipotent stem cells with the extensive renewing capacity to luminal and myoepithelial lineages during neonatal, pubertal, and pregnancy stages. All the adult MaSC population is maintained by signals from their specialized local microenvironment or niche. During puberty, the expansion and maintenance of each lineage are ensured by the presence of two types of lineage-restricted stem cells that can differentiate into either myoepithelial or luminal lineages. Mammary stem cells are self-renewed in their niche. Asymmetrically dividing stem cells produce transit-amplifying daughter cells that commit to basal or luminal progenitor lineages.

The mammary gland is a compound, branched tubuloalveolar structure, and a major characteristic of mammals. The mammary gland evolved from the epidermal apocrine gland, the skin glands as a bilateral accessory reproductive organ located on the ventral surface of the body [1]. Mammary glands produce milk as a source of nutrition for supporting the postnatal survival of offspring for reproductive success in all mammals [2]. Morphologically, mammary glands are formed by several different types of cells. The epithelial cells elaborate the ductal network of the gland and maximize the surface area within a constrained volume, whereas a variety of stromal cells or connective tissues with extracellular matrix (ECM) protein supports the mammary glands. The major components of stromal connective tissues are adipocytes, which constitute the mammary fat pad and retain the embedded ductal network; fibroblasts which support the hematopoietic system; vascular endothelial cells which support the blood vessels; a variety of innate immune cells (both macrophages and mast cells); and nerves [3,4] (Figure 1 and Figure 2). The fatty stroma is the supportive network for the epithelium bi-layered structure and provides nutrients, blood supply, and immune defenses besides the physical structure to the gland. Importantly, each specific stromal cell secretes instructive signals for specific aspects of the development and function of the epithelium [5]. There are two main types of epithelium in the mammary gland, namely, luminal and basal. The luminal epithelium forms the inner layer of the ducts as a laticiferous duct. It surrounds the hollow lumen that differentiates into the milk-producing secretory alveoli or lobules. In contrast, the basal epithelium consists of myoepithelial cells that form the outer layer of mature mammary ducts. It also harbors stem and progenitor cells, which form both luminal and myoepithelial cells/layer [6]. The epithelium ensheathes by one of the main types of ECM, the basement membrane (BM), which separates the epithelium from the stroma and influences the development of the mammary gland [7]. Thus, BMs surround three cell types in the mammary gland, namely, the epithelium, the endothelium of the vasculature, and the adipocytes. In males, mammary glands are present as a rudimentary structure and generally nonfunctional form. We reviewed the recent findings on the molecular journey of mammary gland development from prenatal to lactation.

## 2. Development of Rudimentary Structure of Mammary Gland

The mammary gland develops as a rudimentary structure from a thickening under the ventral skin during embryogenesis. This rudimentary structure grows into a rudimentary branched ductal tree embedded in one end of a larger mammary fat pad at birth. The embryonic development of the mammary gland is a series of several hormone-independent specialized events [8].

### 2.1. Human Rudimentary Structure of the Mammary Gland

The human mammary gland development is initiated during embryonic life from the parenchyma as a single epithelial ectodermal bud. The first visible indication of primitive mammary bud development can be recognized on day 35 (week 5), with the proliferation of paired areas in the epidermis of the thoracic region [9]. Subsequently, two distinct mammary ridges or milk streaks are formed between the fetal axilla and inguinal region. By the end of week six, the mammary ridges become regressed to two areas in the thoracic region (2nd–6th rib). Two solid epithelial masses as mammary bud begin to grow downward into the underlying mesenchyme [9]. The solid epithelial masses evaginate into the underlying mesenchyme and are surrounded by fibroblast-like cells within a dense collagenous stroma. During the seventh and eighth weeks of gestation, the mammary parenchyma invades the stroma, which appears as a raised portion called the mammary disc.

On week 9, the mammary placodes grow inward further as a cone stage. Between the 10th and 12th week, epithelial buds sprout from the invading placodes and transform into globular shapes with the notching of the epithelial–stromal border as a nascent stage. Parenchymal branching occurs during the 13th through 20th weeks. Between the 12th and 16th weeks of gestation, the nipple and areola form in the epidermis and overlie the developing glands with the differentiation of the mesenchymal cells into fibroblasts, smooth muscle cells, capillary endothelial cells, and adipocytes. On week 15 occurs branching of 15–25 solid epithelial cords, called the branching stage. On week 20, the solid mammary cords canalize; the epidermis in the region of the nipple becomes depressed and forms the mammary pit [9]. After that, the cuboidal epithelial cell line forms a bilayer around the ducts. The luminal layer rapidly acquires the characteristics of secretory cells, whereas the basal layer becomes myoepithelial. By six months of gestation, the basic tubular architecture of the fetal glands becomes established. Branching continues, and canalization of the cords occurs, forming the primary milk ducts by 32 weeks gestation [1]. At 32 weeks’ gestation, the ducts open onto the area, which develops into the nipple [10]. The adipose tissue of the mammary gland develops from connective tissue that has lost its capacity to form fibers and support further growth of parenchyma [11]. The fat islands are within a fibrous connective stroma that separates the ducts. The primitive secretory epithelial cells, which become functional near the end of gestation, respond to the lactogenic hormones of pregnancy [9]. In humans, mammary glands develop similarly in female and male fetuses [12]. Due to difficulties in precisely establishing the day of conception, the phases of mammary gland development are correlated with embryonal or fetal size [9] (Table 1).

The newborn mammary glands are a very primitive structure, composed of ducts ending in short ductules or epidermal ridges (milk lines) lined by one to two layers of epithelial and one layer of myoepithelial cells. The epithelial cells have eosinophilic cytoplasm and lipid droplets with typical apocrine secretion and fine cytoplasmic vacuolization. Therefore, colostrum can be expressed from the infant’s mammary glands shortly after birth. This is attributed to the pro-lactation hormones present in the fetal circulation at birth. The secretory activity of the newborn glands subsides within 3–4 weeks [13]. The BM of the mammary bud and early projections contain type IV and VII collagen and laminin-a3, and the epithelium displays β1, β4, and α6 integrin expression [14]. However, BM signaling in the embryonic gland is unknown [14]. Regression of the mammary gland usually occurs by four weeks postpartum and coincides with a decrease in the secretion of prolactin from the anterior pituitary gland of the infant [11]. After birth, the mammary gland becomes quiescent until the onset of puberty. Thus, the ducts in the newborn breast are rudimentary and have small, club-like ends that regress soon after birth.

### 2.2. Mouse Rudimentary Structure of the Mammary Gland

The mouse model combined with tissue recombination techniques was used to generate chimeric glands to understand the morphogenesis and lineage commitment events during embryonic stages of mammary gland development. Moreover, the mouse model supports different stages of development at specific time points in a genetically identical group and supports conducting extensive in vivo studies. The milk or mammary lines formation initiation begins mid-gestation on an embryonic day (E) 10.5 [15]. Within 24–36 h of formation, the mammary line resolves into five pairs of lens-shaped placodes in the mouse. The epithelial placodes are a lens-shaped thickening of surface ectoderm formed by several layers of columnar cells invaginate from the ectoderm. These placodes invade the presumptive mammary mesenchyme to create a naive ductal network [16,17]. On E12.5, each placode expands and invaginates into the underlying mesenchyme to form a mammary bud [18,19]. The mammary epithelial cells proliferate downward and lead to bud growth as mammary sprouts into the dense mesenchyme until it reaches the developing mammary fat pad located within the dermis. The mammary fat pad consists of a loose collection of preadipocytes originating from mesenchymal condensation on E14. The onset of ductal branching, the morphogenesis starts on E16. The mammary fat pad is formed by the skin overlying the primary mammary mesenchyme and is remodeled into a second stromal compartment filled with preadipocytes [16]. At this stage, primary cord or mammary sprouts start dichotomous branching and give rise to the rudimentary ductal tree with a primary duct with 10–15 secondary branches present at birth. Concurrently, a ductal lumen is formed, and the skin overlying the primary mammary mesenchyme remodel into a typical nipple structure. This process involves thickening the epidermis, suppressing the hair follicle development, and invagination of a concentric ring of keratinocytes that forms the nipple sheath [20]. Thus, in female mice, the simple nascent structure is formed by iterative branching and maintains a continuous BM at the epithelial–mesenchymal interface. In contrast, in male mice, testosterone (T) elicits condensation of the mesenchyme around mammary buds and triggers the destruction of the epithelial rudiment by day 16 [21].

Upon completion of embryonic development, both rodents and humans have similar rudimentary mammary parenchymal structures or a superficial branch organotypic epithelial structure as mammary buds. Mammary buds are sphere structures of concentrically arrayed mammary epithelial cells hanging from the skin by a stalk of epidermal-like cells surrounded by condensed mammary mesenchyme [14,16,22].

### 2.3. Regulators of Embryonic Rudimentary Mammary Development

**Figure 3:**
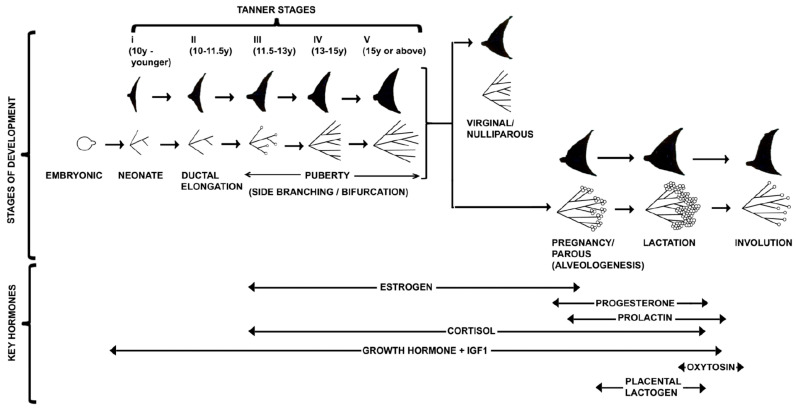
A schematic representation of different stages of mammary gland development connecting with embryonic mammary primordial, followed by the different postnatal stages along with key hormone dependent phases vs. independent phases, key transcription factors, and signaling molecules. The single circle represents terminal end buds (TEBs) during puberty, whereas multiple circles represent lubloalveolar units.

**Figure 4:**
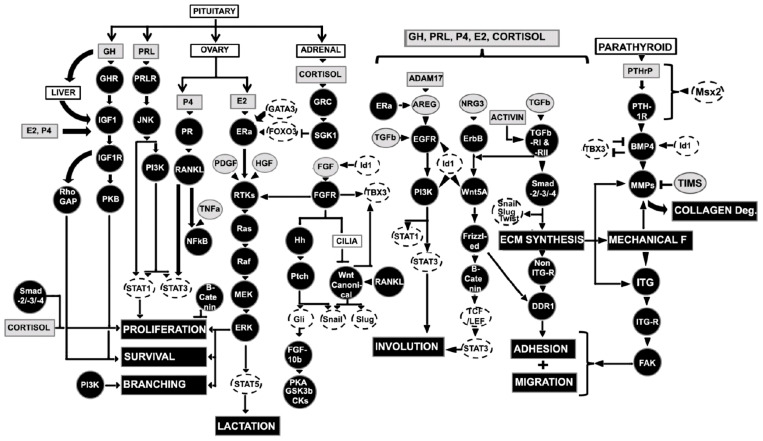
A schematic representation of major signal transduction pathways and their interaction during mammary gland development. Hepatocyte growth factor (HGF), fibroblast growth factor (FGF), and platelet-derived growth factor (PDGF) signal through receptor tyrosine kinases (RTK) activates the central Ras-Raf-MAPK pathway or the PI3K pathway and the Src-STAT pathway. Transforming growth factor (TGF)-β signals through the receptor serine/threonine kinases (RS/TK) activates the central R-Smad/Co-Smad pathway or the PI3K and MAPK pathways or Wnt pathway. Jagged and Delta-like ligands signal through Notch receptors regulates the transcription factor CSL. Wnt ligands signal through Frizzled receptors regulates β-catenin and the transcription factors LEF-1/TCF. All these pathways together regulate gene expressions and that determines EMT. The white circles with dotted lines represent the transcription factors.

The embryonic or prenatal mammary development is regulated by multiple genes and transcriptional and translational products through complex signaling pathways based on mouse model studies and surrogate model systems (in vitro culture in 3D gels composed of extracellular matrix components) (Figure 3 and Figure 4). The initial branching morphogenesis of the embryonic mammary gland is a hormone-independent process [1,23]. Studies have demonstrated that mice have no apparent effects of the growth hormone receptor (GHR), estrogen receptor α/β (ERα/β), prolactin receptor (PRLR), and progesterone receptor (PR) during embryonic mammary development [1,23]. Based on mouse model and mammary cell line studies, wingless-related integration site (Wnt)/β-catenin, fibroblast growth factors (FGF), hedgehog (Hh), insulin-like growth factor-1 (IGF-1), parathyroid hormone-related protein (PTHrP), neuregulin 3 (NRG3), and their receptors are key signaling molecules during embryonic mammary development [16]. These signaling molecules regulate a wide range of transcription factors (TFs) from the Homeobox gene family (HOX), GATA binding protein 3 (GATA3), and the T-box family (TBX) in the endoderm or mesoderm.

Wnt/β-catenin signaling is universally required by all mammary placodes and ectodermal appendages [18]. There are nineteen Wnt ligands, and ten Frizzled receptors are known [24]. *Wnt* ligands act as morphogens that provide positional information to neighboring cells through canonical and non-canonical intracellular signaling [25,26]. In the canonical signaling pathway, the binding of Wnt with a Frizzled receptor and a low-density lipoprotein receptor-related protein 5 or 6 (LPR) causes the disassembly of a multiprotein complex containing glycogen synthase kinase 3 (GSK3), casein kinase 1α (CK1α), axin, and adenomatous polyposis coli (APC). This process supports the translocation of β-catenin into the nucleus and binds lymphoid enhancer-binding factor 1 (LEF1) transcription factor [27]. In the non-canonical signaling, the β-catenin-independent pathway is activated either by the change in cell polarity or cytoskeletal rearrangement through the Planar Cell Polarity (PCP) pathway or the intracellular Wnt/Ca^2+^ pathway [27]. The PCP pathway directs asymmetrical cytoskeletal rearrangement and cellular polarity through disheveled JNK and Rho family GTPase, whereas Wnt/Ca^2+^ pathways, Frizzled acts through G-proteins to activate phospholipase C (PLC), resulting in activation of various transcriptional factors [27]. A partial list of Wnt target genes is documented in mammary tissues, including *Cyclin D1*, c-*Myc*, *Wisp1*, *Wrch1*, *Stra6*, *Stromelysin-1*, *Cox-2*, and *Twist* mammary development [28]. Both loss- and gain-of-function experiments have demonstrated that canonical epithelial and mesenchymal Wnt/β-catenin signaling is critical for the initial mammary lines, placodes, and mature mammary bud development [18,29,30,31]. Disruption of Wnt signaling within the developing epidermis through transgenic expression of the secreted Wnt inhibitor dickkopf-related protein 1 (DKK1) abolish all morphologic evidence of mammary development. DKK1 suppresses early canonical Wnt signaling, subsequent Wnt10b induction along the mammary line, and all placodal growth [18]. In contrast, activation of Wnt signaling results in the accelerated formation of enlarged mammary placodes [18,32]. Several other Wnt isoforms (Wnt3, Wnt6, and Wnt10b) are activators of early β-catenin signaling. Wnt isoforms express diffusely throughout the ectoderm along with the genetic hierarchy of factors, including ventral bone morphogenetic protein-4 (BMP4), dorsal T-box transcription factor-3 (TBX3), neuregulin-3 and somatic fibroblast growth factor-10 (FGF10), and act upstream to define the dorsal–ventral position along the mammary line [8,33]. The whole mount in situ hybridization studies suggested that *Wnt10b* (formerly *Wnt12*) is expressed in the mammary buds, E11/12 to E14/15 [34]. Therefore, Wnt10b regulates canonical Wnt/*β*-catenin signaling in mammary bud development and acts on epithelial or mesenchymal components [28]. As a downstream component from *β*-catenin and Lef1, Wnt signaling promotes the development of mammary rudiments by upregulation of the homeobox genes *Msx1* and *Msx2* [28]. The microarray studies have demonstrated that β-catenin target genes represent an essential module of the PTHrP-induced mammary mesenchyme specification process [35].

Similar to Wnt/β-catenin signaling, FGF signaling is essential to the early stages of mammary development and acts in parallel with Wnt signaling [16]. There are 23 FGF ligand members. However, only a few subsets have been studied in the mammary gland [36,37]. The inhibitor of Wnt signaling does not alter the expression of FGF10 or FGF receptor 1 (FGFR1) [18,32]. FGF10 is expressed in the developing mammary line, whereas FGFR2β is expressed within the developing mammary epithelial placodes. Interestingly, *FGF10* and *FGFR2β* genes knockout mice cannot form four pairs of placodes (number 1, 2, 3, and 5) [38,39]. FGF members signal in a paracrine manner from mesenchymal cells to epithelial cells. FGF binding with FGFR activates downstream signaling including the Ras-Raf-MEK-ERK and PI3K-Akt pathway, resulting in cell survival and proliferation.

Hedgehog (Hh) signaling pathway plays a crucial role in epithelial–mesenchymal interactions, cell differentiation, promoting proliferation, patterning, and survival during embryonic development [40]. Upon stimulation of the Hh pathway, the zinc finger transcription factors Gli2 and Gli3 are activated and promote transcription of the direct target gene Gli1 [41,42]. Knockdown and mutant studies have shown that somatic Gli3 regulates expression of FGF10, which in turn signals to ectodermal FGFR2b and thence, to Wnt10b [38,39]. Gli3 encodes a microtubule-bound transcription factor that phosphorylates to generate a repressor (CiA), or proteolytically cleave to generate a repressor (CiR), which regulates mammary bud formation [43]. Interestingly, GliA/GliR ratio (Gli activator forms to Gli repressor forms) provides a crucial developmental signal threshold for buds 3 and 5 in mice [4].

IGF-1 and insulin-like growth factor receptor (IGF1R) signaling support embryonic mammary bud morphogenesis through RhoGTPase activating (Rho-GAP) family and insulin receptor substrate-1/2 (IRS) effector protein in the epithelial–mesenchymal interactions [44]. IGF-1 is produced in the liver in response to the pituitary growth hormone (GH). Studies have shown that embryos deficient in P190-B, a member of the Rho-GAP family that interacts with integrins, had smaller mammary buds due to the defect in both compartments, mainly by lower proliferation of the epithelial bud and the aberrant underlying mesenchyme (45). A similar phenotype was observed in embryonic mammary buds lacking IRS-1/2 [45].

PTHrP and PTH1R signaling from epithelium to mesenchyme supports the formation of a rudimentary ductal tree, nipple, and nipple sheath and determines epithelial cell fate [4,46]. PTHrP is secreted by mammary epithelial cells and sensitize mammary mesenchymal cells [46], whereas PTH1R is expressed in the mesenchyme underlying the developing bud. The disruption of either the PTH1R or PTHrP gene in mice fails the rudimentary ductal tree and formation of the nipple [20,47]. An autocrine BMP4 acts as a downstream factor that triggers PTHrP signaling to support ductal outgrowth in the mammary mesenchyme [35,46]. Other studies have demonstrated that the treatment of mice in a combination of BMP4 and PTHrP enhance matrix metalloproteinase 2 (MMP2) activities in mesenchymal cells. In contrast, MMP inhibitors block PTHrP dependent mammary bud outgrowth in culture [35]. PTHrP signaling is acts to sensitize the primary mesenchyme to pre-existing BMP4 expression in the ventral dermis. This process induces the expression of a subset of specific genes such as Msx2 transcription factor and MMP2 in the mesenchyme, which mediates the various morphogenetic tasks in initiating ductal morphogenesis from the bud [8]. Similarly, overexpression of PTHrP in basal keratinocyte converts dermis to mammary mesenchyme and suppresses hair follicle formation [20], and ultimately, supports mammary gland development. Ablation of epithelial SHH signals transforms mice hair follicles into a mammary-gland-like structure [48].

NRG3, a member of the epidermal growth factor (EGF) family, is critical for embryonic placode formation by augmenting or facilitating Wnt signaling [49]. NRG3 and its receptor ErbB4 are first expressed in the lateral plate mesoderm underlying the ectoderm where the mammary buds subsequently develop, immediately prior to the sequential development of each bud or placode, and promote mammary morphogenesis [49,50,51]. Once NRG3 binds to ErbB, ErbB dimerizes with another ErbB monomer or with one of three related receptors—ErbB2, ErbB3, or ErbB4, in order to exert its downstream effects to support normal embryonic placode formation [51,52]. Studies have shown that EGFs act as a mitogen for both epithelial and stromal cells [53].

### 2.4. Transcriptional Factors

Multiple transcription factors are involved in placode formation and mammary ductal outgrowth, including Gli. A T-box-containing transcription factor (T-box) is essential for placode formation. T-box3 (TBX3) is expressed in the mammary line and developing placodes. Lacking TBX3 in mice fails to produce mammary placodes 1, 3, 4, and 5 and fails to express the placodal markers Wnt10b and lymphoid enhancer-binding factor 1 (Lef-1) [54]. In mice, Lef1 is normally expressed in the epithelial cells of the mammary buds at E11/12 and subsequently induces the condensation of mesenchymes that surround each bud by E14/15 [20]. Induction of Lef1 expression depends on paracrine signaling from the mammary epithelium to the mesenchyme mediated by PTHrP and PTHR1 [20]. In humans, Tbx3 haploinsufficiency is associated with the ulnar-mammary syndrome and severe mammary hypoplasia, and sometimes complete loss of mammary glands [55]. TBX3 expression within the mammary line depends on both FGF and Wnt signaling. TBX3 expression up-regulates the expression of Wnt and FGF signaling pathways for complete mammary line development and transition to placode formation. Thus, TBX3 is downstream and upstream of Wnt and FGF signaling as a paradigm for T-box [16]. The orientation of ectodermal cells, mammary line specification, and placode formation is regulated conversely by bone morphogenic protein 4 (BMP4), which negatively regulates TBX3. Other homeodomain-containing transcription factors, including Msx1 and Msx2, are expressed differentially in the epithelium. Interestingly, Msx2 is expressed only in mammary buds’ mesenchyme [46,56,57]. Interestingly, the loss of either *Msx1* or *Msx2* alone does not affect the formation of the mammary buds, whereas loss of Msx2 affects nipple formation and bud outgrowth [46,57]. Another homeodomain-containing transcription factor, Hoxc6, along with Msx2 are required for mammary ductal outgrowth, since, just before ductal sprouting, Msx2 is expressed in the mammary mesenchyme due to PTHrP/BMP4 signaling [46]. Ultimately, Hox gene regulates epithelial mammary bud regulatory elements (MBRE). However, the complete signaling network during embryonic mammary development is incompletely understood.

These complex signaling networks regulate the epithelial-to-mesenchymal transition (EMT) and support the development of the mammary gland. In addition, epithelial cells lose polarity and adhesion to become mesenchymal cells with migration and invasion properties.

## 3. Journey of Pubertal Mammary Gland

After birth, the rudimentary mammary structure (ductal Anlagen) enters a phase of morphogenetic quiescence. However, the rudimentary mammary structure grows isometrically to the rest of the body and keeps up with normal body growth until puberty. At this stage, the female rudimentary mammary gland development is indistinguishable from the male breast [5]. In males, the onset of puberty androgen-mediated condensation of mesenchyme around the primary ducts results in the elimination of the ducts and the prevention of mammary epithelial growth [58]. In females, the onset of puberty (8–12 years) with the production of steroid hormones (mainly estrogen, E2) from ovaries and growth hormone (GH) from the pituitary along with other systemic factors promote a series of coordinated events that transform the rudimentary mammary structure into the mammary gland. The allometric growth of the mammary gland is due to ductal elongation and secondary branching with both stromal and epithelial growth. This development process is faster than general body growth. At puberty, the increase in breast size is mainly caused by the increased deposition of adipose tissue within the gland. However, progressive elongation and branching of the ducts create a more extensive ductal network [59]. An important aspect of ductal morphogenesis is the patterning, which involves four distinct mechanisms: (a) a bifurcator, which controls endbud splitting; (b) a periodic device, which determines how far apart the branches grow; (c) a restriction collar, which causes the growing epithelium to form a tube rather than a ball; and (d) negative feedback to prevent ducts from colliding [3]. The process of initial specification, formation, and in-growth is called anlage [5]. Thus, the ducts grow into a pre-existing stromal mammary “fat pad”, forming long, thin tubes that are extensively branched. New ducts largely develop from their tips, which are enlarged multicellular structures called “endbuds”. This distinct multilayered bulbous or club-shaped epithelial structure of the mammary gland is known as terminal end buds (TEBs). The tips of the ducts elongate and penetrate further into the fat pad, thus forming the complete branch epithelium tree as the main ductal system. Once the ducts reach the margins of the mammary fat pad, they regress [5,17], leaving behind blunt-ended ductal termini or smaller rounded buds (Table 2, Figure 1 and Figure 3). TEBs are described in primates, including humans and rodents (rats and mice) except ruminants. Ruminants have terminal ductal units (TDU), which direct the elongation and branching of ducts during puberty and resemble a multi-lobular TEB, with each ductule growing from a central chord of epithelial cells typically 4–5 cells thick [60] (Figure 1, Figure 3 and Figure 4).

Each mammary duct terminates in a single bulbous terminal end bud in the pubertal mouse at 4–5 weeks of age. The ducts develop and spread throughout the fat pad in the adult virgin mouse, and the end buds are entirely lost; although there is enough space present between the branched ducts. In contrast to human mammary glands, the mouse mammary gland has significant fat with small amounts of fibrous connective tissues. Interestingly, mouse mammary ducts contain epithelial cells that surround the lumen of the ducts. Like women, mouse luminal and myoepithelial cells express cytokeratins and actin. The myoepithelial cells form a basal layer as a laminin-containing basement membrane and separate the parenchymal and stromal compartments.

### 3.1. Terminal End Buds (TEB)

The TEBs are bulbous and unique to peri-pubertal, highly proliferative, and hormone-dependent structures that grow at the end of growing ducts [12,61,62]. TEBs have two morphologically distinct cellular compartments. The outer compartment (distal surface) is a highly proliferative single-cell layer of “cap cells”, whereas the inner layer of cells surrounds the centrally located “body cells” [63] (Figure 1B). The cap cells give rise to basal cells (basal lamina), interact with the surrounding stroma, give rise to the myoepithelial cells enveloping the mature ducts, and are a reservoir for regenerative mammary stem cells [64,65]. Cap cells express several markers for basal lineage, including keratin 5 and 14, smooth muscle actin, p63, and stem cell-specific isoform of SH2-containing inositol 5′-phosphatase (sSHIP) [64]. Body cells from the inner mass of the TEBs give rise to the luminal cells lining the duct’s interior and the presence of ductal and alveolar progenitors [66]. The innermost body cells are incompletely polarized, while body cells adjacent to the basal layer are polarized and form adhering-based adherens junctions. In contrast, cells in the interior are loosely held together with desmosomes [67]. The body cell layer has a high apoptotic index, supporting lumen formation [68]. Alveolar buds are present at the ends of TEBs during pregnancy and are responsible for milk production. Alveolar buds are the precursors of the secretory units called alveoli. Alveoli are a few millimeters in size, lined by one to two layers of cuboidal epithelium, surrounded by the myoepithelial cells, and have well-defined lumina.

During the periodic estrus (rodents) or menstrual (human) cycle, the alveoli undergo cyclic expansion and maturation, followed by a modest regression phase as ovarian hormone levels rise and fall, respectively [69,70]. With repeated menstrual cycles, the rudimentary gland transforms into a complex ductal network by bifurcation of the TEBs and secondary side branches that sprout laterally from the trailing ducts until the entire fat pad is filled with a network of branched ducts [5,36]. From the end of the tertiary branches, tubuloalveolar structures start forming. The lobules are relatively quiescent in the first half or follicular phase of the menstrual cycle. At this stage, lobules are small, with few alveoli, and there is low mitotic activity. During the luteal phase (after ovulation), the lobules and alveoli develop with open lumens and the highest mitotic activity [71]. Therefore, at the premenstrual phase, women commonly experience a sense of fullness due to the highest cell proliferation with vacuolization of epithelial cells that lead to edematous stroma [72]. The open architecture of the ductal network depends on the distance maintained from each other and epithelial cells in the ducts. Presumably, distinct mechanisms control the timing of endbud branching during the growth of major ducts and the periodicity of side branch eruption from pre-existing main ducts. Side branches appear intermittently along ducts, particularly during estrus/menstrual cycles and in pregnancy, and are the precursors of alveoli [73].

Ultimately, the development of TBE is regulated by several cell types orchestrated together with mechanical cues and cellular rearrangements, which finally established the pattern of the mammary gland [74]. The morphogenetic changes of the mammary gland during development are tightly regulated by the proliferation of progenitor cells, the differentiation programs, and the maintenance of tissue homeostasis, which controls turnover of cells (apoptosis) through steroid and peptide hormones, growth factors (GFs), receptor tyrosine kinases, extracellular matrix (ECM), and proteases [75].

### 3.2. Extracellular Matrix (ECM) 

The mechanical properties of ECM support the stromal–epithelial interactions and pattern formation during the morphogenesis of the mammary gland. ECM determines the complex ductal network of TEBs and secondary side branching during ductal morphogenesis [76,77]. ECM is synthesized and secreted by epithelial, myoepithelial, endothelial, immune cells, fibroblasts, and adipocytes [78,79,80,81]. ECM interacts through numerous weak non-covalent bonds and crosslinking via covalent bonds for scaffold organization, tensional characteristics, and stabilization [81]. ECM accumulates at the cleft of endbuds (bifurcation) and supports a wedge to split growth into a new direction. ECM density (stiffness) influences epithelial cell fate decisions through cell adhesion, survival or death, polarity, proliferation, differentiation, and lineage of stem cell progenitors [82].

#### Biochemical Composition of ECM

ECM is thin, ~100-nm thick sheets of glycoproteins and proteoglycans. The glycoproteins and proteoglycans are polymers of laminins (LM) and a cross-linked network of collagen IV fibrils [83]. LM is heterotrimeric (αβγ trimers) glycoprotein (~900 kDa) with 15 different combinations that are derived from five a, three β and three γ subunits, and coded from distinct genes [84]. In the mammary gland, four distinct LM isoforms [laminin-111 (LM-1; α1, β1, γ1), -322 (LM-5; α3, β3, γ2), -511 (LM-10) and -521 (LM-11)] are present [85,86]. Similarly, BM proteoglycans are complex glycosaminoglycans (GAG) chains that vary with developmental stages of the mammary gland [87]. However, the major component of BM is perlecans. BM interacts with mammary–epithelial cells (MEC) through integrins and transmembrane (TM) proteoglycans, dystroglycan, and syndecan, and is joined to the cytoskeleton for the signaling platform, and controls cell fate [88,89]. Epithelial cells receive instructive signals from the ECM through different receptors, including β1-integrin (collagen receptor) and non-integrin receptors discoidin domain receptor 1 (DDR1, recognize multiple ECM proteins) [90], dystroglycan, and syndecan [91]. Integrins are TM receptors and are well studied in MEC. Structurally, integrins are composed of 18α and 8β subunits as a heterodimer and form 24 canonical integrin receptors [92,93]. These canonical integrin receptors are for collagen (α1β1 and α2β1), LM-111, -511, -521 (α3β1, α6β1, and α6β4), LM-322 (α3β1 and α6β4), MEC fibronectin, and vitronectin (α5β1 and β3 integrins) [94]. The terms BM and basal lamina have been used interchangeably to refer to the highly specialized ECM, which organizes 20–100 nm thick structure directly underlying the epithelium [81]. The other major constituents of the basal lamina are LM, collagen IV, nidogens, and perlecan which act as adhesive contacts in epithelial cells. Collagen IV is a heterotrimer formed from six genetically distinct chains. Collagen IV provides an anchor for mammary epithelial cell viability. Nidogen (entactin) is a sulfated glycoprotein (150 kDa), synthesized by fibroblast, and helps in the formation of the basal lamina. Perlecan is a proteoglycan that consists of a core protein (470 kDa) with covalently linked heparin sulfate, a specific class of GAG, and increases the molecular weight of perlecan over 800 kDa [95]. Perlecan provides instructive cues and is dependent on the context and structural integrity of ECM. Therefore, ECM serves as a major reservoir for GFs and cytokines and alters according to tensional requirements and organization, which change throughout to generate the open architecture of the mammary gland development [74].

### 3.3. Stroma

The mammary stroma supports proper ductal elongation and branching morphogenesis. The stroma is adjacent to the basal lamina (BM), a 10–100 μM thick band of an organized collagen-rich structure known as the intralobular stroma. The stroma surrounds individual alveoli or endbuds, which remodel as the cells collectively invade the intralobular stroma [81]. Bands of stroma surrounds a cluster of alveoli that forms a single lobule, referred to as the interlobular stroma. The mammary stromal cell type includes adipocytes, fibroblasts, macrophages, eosinophils, neutrophils, and endothelial cells, primarily solitary and embedded within the fibrous ECM [10]. Thus, the stromal ECM influences cell-matrix interactions within the mammary epithelium and controls ductal development and alveolar functions. Stromal ECM components include fibrillar collagens, proteoglycans, hyaluronic acid, fibronectin (FN), and tenacins. However, the composition varies with the developmental stages of the mammary gland during pregnancy [96]. During duct formation and alveolar expansion, the dominant structural components are fibrillar collagens type I, III, and IV with other ECM, which regulate intra- and inter-lobular stroma composition and functions [81]. The TEBs directly contact stromal cells during ductal elongation [97,98,99,100].

FN is a dimeric glycoprotein (~500 kDa) encoded by a highly conserved gene and supports regulatory protein for branching ductal outgrowth and increases the formation of TEBs [101,102,103]. The fibronectin fibrils accumulate at the clefts between new buds and support branching [101,104].

There are several ECM proteins, including tenascins (TN), secreted protein acidic and rich in cysteine (SPARC/osteonectin), small leucine-rich proteoglycan (SLRP), decorin, and biglycan, along with fibrous connective tissues and elastic fibers that support the cleft formation between new buds and branching [81]. SPARC is a 32 kDa glycoprotein, secretes in ECM, and supports cell–matrix interactions through FN assembly and integrin-linked kinase activity [105,106]. SLRP is an N-terminal cysteine-rich motif with a tandem leucine-rich repeats (LRR) core protein attached with one or more GAG chains. SLRPs bind to cell surface receptors and GFs and support signal transduction pathways [107]. Decorin has a core protein (~38 kDa) linked covalently with single chondroitin sulfate (CS) or dermatan sulfate (DS) chain resulting in 90–140 kDa secreted protein and is expressed in fibroblasts and astrocytes [81,108,109]. Decorin has a pivotal role in the spatial alignment of stromal collagen fibers and inhibits EGFR signaling by initiating EGFR internalization and degradation by caveolar endocytosis [109]. Biglycan is a member of the SLRP class I family with a core protein (~38 kDa) and covalently attached to two GAG chains (chondroitin sulfate and/or dermatan sulfate) with an overall MW of 150–240 kDa [110]. Decorin and biglycan have distinct, non-overlapping roles [111]. Biglycan can induce elastic fiber-associated protein fibrillin-1 expressions that subsequently retain elastic properties of the tissue [112].

Other structurally distinct stromal components are acellular fibrous connective tissue, found in large volumes in both rodents and the human mammary gland. These tissues are characterized by the presence of elastic fibers, fibroblasts, immune cells, and high fibrillar collagen content except the epithelium [81]. The fibrous connective tissue is separated from the intra- and inter-lobular stroma and shares ECM proteins, including fibrillar collagens, FN, TN, SLRPs, and SPARC. Elastic fibers comprise a 31 kDa secreted protein called microfibril-associated glycoprotein (MAGP) and a 350 kDa fibrillin glycoprotein assembled into 10–12-nm microfibril that provides elasticity structural support to the mammary gland [113]. These microfibrils are associated with other elastic fibers, including elastin, fibulins, and proteoglycans, forming MAGP/fibrillin microfibrils that align parallel to fibroblasts. Ultimately, microfibrils support tropoelastin deposition (70 kDa) as a soluble precursor of elastin. Tropoelastin is rich in hydrophobic amino acids and contains a low amount of polar amino acids, including several lysine derivatives. The lysine derivatives are imperative for covalent crosslinking between monomeric elastin via lysyl oxidase (LOX) [81,113]. An additional constituent of elastic fibers is the 50–200 kDa fibulin family protein. Fibulin proteins have calcium-binding sites and consist of I, II, III domains with EGF-like fragments in their central segment. Fibulin 5, a 66 kDa, has a strong calcium-dependent binding to tropoelastin and weak binding to the carboxyl-terminal of fibrillin 1. The proteoglycans, mainly biglycan and decorin, have been implicated in elastogenesis through binding to tropoelastin, fibrillin-1, and MAGP [112,114,115].

#### 3.3.1. Glycosaminoglycans (GAG)

Various classes of GAGs are associated with a distinct role in ductal elongation [81]. GAGs are anionic linear polysaccharides with repeating hexuronic acid. The hexosamine binds to GFs and cytokines in ECM [81]. GAG act as “glue” that allows GFs to “stick” to the basal lamina and fibrillar ECM proteins. Electron microscopy has shown that the BM that surrounds the tips of end buds in mice are thin and rich in hyaluronic acid, while the BM along the flank of end buds is thicker and associated with sulfated GAGs [116]. Release and activation of GFs and cytokines from ECM can alter matrix stiffness and induce ECM proteolysis [117,118].

#### 3.3.2. Actin and Tubulin

The network of actin and tubulin supports cytoskeletal polymerization at the peripheral end buds and promotes protrusive force in ducts [119,120]. Actin is a ~42-kDa globular protein with three isoforms (α, β, and γ); whereas tubulin is a ~55 kDa globular protein with two isoforms (α- and β-). Both actin and tubulin support the trafficking of integrins through early endosomes and the formation of new matrix adhesions and controls cell migration [121].

#### 3.3.3. Lysyl Oxidase (LOX)

LOX, a copper-dependent enzyme, catalyzes intra- and inter-molecular crosslinkers of fibrillar collagens and elastic fibers through oxidative deamination of lysine residues [81,122]. LOX activity promotes mammary tissue stiffness, fibrosis, and modulates elastic properties of elastic fibers to establish tissue tension [81,122]. Tissue transglutaminase 2 (tTG2) has also been implicated in crosslinking ECM proteins in the mammary stroma. The crosslinking activity of tTG2 depends on calcium and is supported by catalyzing covalent bonds between glutamine and ε-amino groups of lysine residues [123].

#### 3.3.4. Cadherins

Cadherins are calcium-dependent type-1 transmembrane adhesion proteins. Epithelia are strongly cohesive via cadherin contacts, whereas individual cells that migrate into the stroma are deleted by apoptosis via matrix interaction changes from BM to collagen [124]. The spatial orientation of luminal and myoepithelial cells is controlled by differential adhesivity among the cells. Both luminal and myoepithelial cells express the desmosomal cadherins (Dsg2/Dsc2), whereas the myoepithelial cells express desmosomal cadherins Dsg3/Dsc3. The luminal cells are intrinsically more adhesive and thereby restrict the myoepithelial cells to more external and basal locations as an organization. The adhesive properties are inhibited without the Dsg3/Dsc3 function [125]. The BM matrix supports the bilayered organization along with laminin-111 produced by myoepithelial cells. Interestingly, the myoepithelial cells contain hemidesmosomes, which rivet the cells to BM [126,127].

#### 3.3.5. Integrins

Integrins are receptors that support and coordinate cell signaling through the ECM. Integrin-associated scaffold proteins, including filamin in mammary tissue, detect mechanical cues within the ECM, regulate cell shape, motility, and cell cycle, and control morphogenesis [128,129]. β1-integrins are required to maintain mammary stem cells and help mammary ductal cells to proliferate endbuds [126,130]. The β1-integrins activation influences the expression of FGF receptors (FGFR) [131]. In contrast, FGFR is required for LM-a5 expression and supports a positive feedback loop for the maintenance of FGFR [131]. The BM proteoglycans regulate the delivery of GFs and cytokines and act as a reservoir for GF receptors to control the transfer of GFs across the BM and epithelium [132]. Integrins also regulate c-met (MET or MNNG HOS Transforming gene) signaling by sensing laminin molecules within the BM and allow MET signaling to control morphogenesis [133]. Netrin-1, a laminin-related protein, helps in mammary morphogenesis by controlling cell survival and migration of cells [81,134,135,136].

#### 3.3.6. Adipocytes and Fibroblasts

Adipocytes are the largest population of cells within the fat pad in the mammary gland. In addition to ECM, adipocytes provide the structural environment for the epithelium branching and function and physical support to the immune, lymphatic, and vascular systems. In women, the fibrous connective tissue ratio to adipose tissue is inversed [81]. There is a predominance of fibrous connective tissue between ducts and decreased adipose content [81]. During puberty, mammary adipocytes support formation and branching of TEBs [137,138,139]. Adipocytes secrete adipokines, promote the expression of ERα, IGF1, and HGF within the stroma, and promote secondary and tertiary mammary duct branching. At puberty, adipocytes synthesize and secrete vascular endothelial growth factors (VEGF) as an inducer of vascular growth and support mammary branching [1].

Fibroblasts are spindle-shaped cells distributed around the TEBs. Fibroblasts are biosynthetically quiescent. Fibroblasts secrete ECM macromolecules, proteolytic enzymes, fibroblast growth factors (FGFs), hepatocyte growth factors (HGFs), insulin-like growth factors (IGF-1), cytokines, and chemokines under the influence of estrogen (E2), growth hormones, and extracellular cues to support contraction of connective tissue [99,140,141]. Moreover, fibroblasts maintain ECM synthesis and degradation by producing laminin, collagen, fibronectin, proteoglycans, MMPs, and TIMPS [99].

#### 3.3.7. Macrophages and Eosinophils

In the stroma, macrophages are localized to collagen fibers and support the synthesis of long collagen fibers in ECM around the neck region of the TEBs and promote ductal elongation and branching [98]. Macrophages are recruited by producing colony-stimulating factor 1 (CSF1) by epithelial cells around the neck region of TEBs. Macrophages are also present within the body cell layer and support lumen formation by clearing apoptotic cells via phagocytosis [97,98]. Interestingly, macrophages in the stromal compartment of the mammary gland maintain epithelial stem cells as epithelial progenitor cells in an undifferentiated state and maintain TEBs numbers [142]. Mast cells, which are effectors of the innate immune system, also surround the TEBs during puberty [143,144] and induce branching by secreting serine proteases [144,145].

Eosinophils are recruited by the secretion of eotaxin by TEBs and surround the TEBs [97,99]. E2 and P4 dependent amphiregulin secretion by TEBs promotes eotaxin secretion and promotes branching [97,99,146]. Eosinophils secrete different cytokines and growth factors, including VEGF [147]. Ultimately, both macrophages and eosinophils spread throughout TEBs and support pubertal remodeling of the mammary gland through cellular renewal and formation of lumen of the ducts.

### 3.4. Basement Membrane (BM)

The cap and myoepithelial cells deposit the BM during ductal elongation, which results in both polarization of the luminal layer and geometric confinement of the subtending duct. Laminin-1 is expressed by myoepithelial cells and supports the polarization of luminal cells [116,148,149]. The tip of TEB covers in hyaluronic acid and laminin. In contrast, BM in the neck of TEB is a meshwork of collagen IV, laminin-1 and 5, and heparan sulfate proteoglycan [77].

### 3.5. Role of Hormones and Growth Factors in Regulation of Pubertal Mammary Gland

The morphogenesis of the female rudimentary mammary gland up to puberty is a hormone-independent process. Although various hormone receptors are expressed before puberty, the fetus is exposed to high maternal and placental hormones [1,150,151]. At the beginning of puberty, the rudimentary mammary epithelium has asymmetric branched geometry and patterning information encoded in the pre-existing non-spherical structure. The onset of puberty, the ductal elongation and branching of the mammary gland are regulated by many hypothalamic–pituitary–ovarian hormones [5,44,152]. During pubertal mammary development, various hormonal signals are at nano- or pico-molar concentration, amplifying through temporal and spatial autocrine-paracrine signaling molecules and transcriptional coactivators that express and diffuse as a morphogen differentially in TEBs and integrated between the epithelium and stroma. Ultimately, these signaling molecules trigger TEBs proliferation and bifurcation in a controlled manner [74].

#### 3.5.1. Estrogen (E2)

Estrogen is the critical regulator of branching in the pubertal mammary gland. E2, the female sex steroid hormone, is synthesized and secreted primarily by granulosa cells of developing follicles in the ovary during the estrous and menstrual cycle [4,153,154]. E2 is also synthesized locally by adipose tissue in the mammary gland. E2 signaling is mediated by two receptors, ERα and ERβ, in 30% of mammary luminal epithelium, while basal cells do not express ER-receptors [153,155,156,157]. Both humans and rodents have ERα and ERβ have a similar sequence homology and binding affinity for E2. Studies have demonstrated that loss of ERα signaling causes a deleterious mammary phenotype and impaired function, whereas ERβ loss does not. The multiple regulatory elements regulate the ERα gene (ESR1) expression, including transcription factors, chromatin environment, autocrine, paracrine, and endocrine secreted factors and multiple environmental factors (cell–cell and matrix interactions, mechanical forces) [153]. The ERα receptor has six structural domains along with two sub-domains, namely, a ligand-independent (AF-1) and a ligand-dependent (AF-2) subdomain. The AF-1 domain is transactivated and phosphorylated at serine 104/106, 118, or 167 by kinases in response to growth factors including epidermal growth factor (EGF), insulin-like growth factor-1 (IGF-1), tumor growth factor (TGFα).

The presence of ERα in the stroma is not required for mammary gland development [158]. In contrast, ERα is required for TEBs and ducts development inthe fat pad [158], including prepubertal growth, alveologenesis, and lactation during late pregnancy [159]. Ultimately, E2 through ER supports TEBs proliferation with ductal elongation and clefting (bifurcation) of the ducts to generate branches. E2 is a dominant regulator of epithelial cell proliferation. However, E2 has synergistic effects on epithelial and stromal cells along with local growth factors. E2, upon binding with ERα in luminal cells, promotes multiple signaling pathways including genomic action through binding to DNA directly or indirectly (tethering) by physically interacting with other transcription factors including stimulating protein 1 (SP1), activator protein 1 (AP-1), signal transducer, and activator of transcription 3 (STAT3) and Nuclear factor–κβ (NF-κβ), and by a generation of second-messenger molecules (cAMP-dependent signal transduction pathway in the cytoplasm [153,160,161]. The nongenomic action of ER-signaling includes crosstalk with growth factor receptors and G-protein coupled receptors in the cytosol. ERα can activate Src-kinase, leading to epidermal growth factor receptor (EGFR), mitogen-activated protein kinase (MAPK), and phosphatidylinositol-3-kinase signaling [153,162,163,164,165]. E2 through ERα in luminal cells promote amphiregulin (Areg) expression as a transmembrane precursor and are cleaved by ADAM17 to stromal cells [166,167,168]. Areg signals as a paracrine factor to the stroma through EGFR and promotes additional growth factors, including stromal FGF [169]. FGF binds with epithelial FGFR2 and promotes epithelial cell proliferation. E2 also supports FN expression in the mammary gland [170]. FN protein level is increased up to three folds in the mammary epithelium in pre-puberty and sexual maturity [170]. Moreover, mouse models revealed that the transcriptional activity of ERα depends on its interaction with coregulators. In this process, GATA3 regulates FOXA1, which in turn regulates ERα, while GATA-3 and ERα regulate each other positively in the mammary epithelium, the balance between the basal and the luminal lineages and altered mammary development [153].

The mammary fat pad is highly vascularized during lactogenesis and lactation for transporting fluids and nutrients. Interestingly, the VEGF promoter contains an E2 response element [171] that permits transcription of the VEGF gene in cells expressing ERα upon binding of E2 [172]. Thus, during puberty, ovarian E2 also induces communication between endothelial cells within blood vessels, epithelium, and adipocytes.

#### 3.5.2. Growth Hormone (GH) and Insulin like Growth Factor-1 (IGF-1)

Growth Hormone (GH, somatotropin) is a single chain polypeptide (191-amino acid) that promotes the development of TEBs and ductal branching [173]. GH is synthesized, stored, and secreted by somatotropic cells of the anterior pituitary gland. GH acts on mammary development through GH-receptor (GHR). GHR homodimerizes upon GH binding and activates the cytoplasmic tyrosine kinase, JAK2 [174,175]. Subsequently, JAK2 activates three major signaling systems in response to GH, namely, transducers and activators of transcription (STATs, mainly STAT5b), phosphatidylinositol 3-kinase (PI3K), and extracellular signal-regulated kinase (ERK) [176,177,178]. STAT5b activation triggers the transcriptional activity of GH targeted genes [177]. Several molecular signaling pathways coupling GH to ERK are activated, including the SHC-Grb2-Sos-Ras-Raf pathway, EGFR, insulin receptor substrate-1 (IRS-1), Gab-1 tyrosine phosphorylation, and SHP-2 tyrosine phosphatase activity (Frank, 2008). ERK is critical for GH-induced c-fos transcriptional regulation, promoting proliferation and crosstalk with EGF signaling [177].

IGF-I plays an essential role for the GH/IGF axis as a paracrine factor for the growth and development of the mammary gland [141,152,153,158,179,180]. In response to GH, IGF-I is mainly synthesized in the liver and acts on the mammary fat pad, although IGF-I is synthesized in mammary stromal fibroblasts [181,182,183]. IGF-I acts through IGF-IR, which are predominantly epithelium cells. The complete action of GH on mammary development is mediated by IGF-I [44]. Interestingly, both E2 and P are dependent upon IGF-I for their actions, as with several other growth factors [44]. E2 enhances the action of IGF-I through a stromal epithelial interaction and pubertal mammary development [141,184]. The IGF-I-PKB axis is an important survival path for the proliferation and differentiation of TEBs [185]. Thus GH, IGF-I, ovarian E2, and P4 and their respective receptors are important in the post-pubertal branching morphogenesis of TEBs.

#### 3.5.3. Wnt, Hedgehog (Hh), and Fibroblast Growth Factor (FGF) Signaling

Wnt and Hh signaling are coordinated by primary cilia present on mammary epithelial cells during puberty and epithelial plasticity [186,187,188]. Hh signaling is involved in tissue homeostasis, regeneration, and stem cell maintenance of the pubertal mammary gland [189]. Upon Hh activation, the primary cilia serve as the processing sites for Gli transcription factors. They are involved in a multi-protein complex consisting of a subset of intraflagellar transporter proteins, protein kinase A (PKA), glycogen synthase kinase 3β (GSK3β), casein kinase (CK), etc. [190,191]. Studies have shown that the suppressor of fused (Sufu) plays the role of a negative regulator of Hh/Gli signaling [192].

Both canonical and non-canonical Wnt pathways are activated during mammary cell fate determination, maintenance of mammary progenitor cell populations, side branching morphogenesis, and alveogenesis [28,193,194]. The ligands (Wnt4 and Wnt5a) and non-canonical receptors are localized to the luminal compartment, whereas Wnt6 is localized to the basal layer [195]. Primary cilia are regulated by FGFs in epithelial tissues and interact with growth factors (GFs), WNT, and Hh signaling during mammary morphogenesis [196]. Another component of Wnt signaling is a transcription factor LEF1, requiring the development of mammary rudiments [197]. GH and IGF1 induce local Wnt expression and Frizzled family of Wnt receptors in the TEBs to support the proliferation of the mammary gland [28,193,198]. Interestingly, the serine/threonine phosphorylation of the N-terminal domain of β-catenin by GSK3β and CK1 kinases play a significant role in the compartmentalization of β-catenin [81]. In the normal mammary gland, the majority of β-catenin localizes to cell–cell adherent junctions through association with cadherins. The cadherin/catenin complexes are critical to mammary integrity. The cytosolic and nuclear β-catenin transduces signals from multiple pathways into cell-context-specific gene expressions essential for mammary development [33]. However, the detailed Wnt signaling pathway is not well studied in pubertal TEBs development.

The FGF1, FGF2, FGF4, FGF7, and FGF10 are highly expressed during mammary ductal elongation [104,199]. FGF7 has an inhibitory role in branching and inverse relation with TGFα signaling [104]. FGF10 produces by adipose tissues. FGF10 is localized in mesenchyme near ducts and alveoli and acts on TEBs epithelium through FGFR2b in a paracrine manner [200,201]. Recent studies suggest that FGF and its receptors maintain the basal compartment for regeneration [37,202].

#### 3.5.4. Epidermal Growth Factors Signaling

Epidermal growth factors (EGF) and epidermal growth factor receptors (EGFRs) act as an integrated paracrine factor in the epithelium and stroma during pubertal ductal growth and branching of TEBs [177,203]. EGF family ligands are expressed as transmembrane precursors, cleave enzymatically (ADAM and MMPs), and bind with the receptors. Subsequently, receptors dimerized and activated intracellular kinases through phosphorylation [204,205,206,207,208,209,210]. The ligand neuregulin (NRG1–4) and ErbB receptors (EGFR/HER1, ErbB2/HER2, ErbB3/HER3, and ErbB4/HER4) contribute to the proliferation and survival of mammary epithelial cells [204,205,208]. ErbB2/HER2 and ErbB3/HER3 are highly expressed in the epithelium, but EGFR or ErbB4/HER4 is required only in the stroma [52,206,209]. Interestingly, EGFR and ErbB2 appear most prominently involved in ductal morphogenesis in puberty. Activating the tyrosine kinase in ErbB receptors results in the phosphorylation of multiple tyrosine residues within their intracellular domains (210). Phosphorylation at ten tyrosine residues of EGFR [211,212,213] leads to the recruitment of many docking and signaling proteins, including Grb-2, SHC, PTP-1B, SHP-1, SHP-2, Cbl, and PLC-γ [214,215] that influences several downstream signaling pathways. Moreover, cholinergic stimulation through parasympathetic innervation triggers epithelial cells to release heparin-binding EGF and promotes branching morphogenesis by EGFR [142,216,217]. Ultimately, downstream signaling pathways of ErbB receptors regulate gene expression and cell behavior. The ductal development proceeds only when EGFR phosphorylation occurs through the transmembrane zinc-dependent cell surface disintegrin and metalloproteinase domain-containing protein 17 (ADAM17, TNFa converting enzyme, TACE) with AREG expression on mammary epithelial cells and EGFR expression in stromal cells [168,203,205,209,218,219]. ADAM17 is a family of zinc-dependent cell-surface enzymes and is responsible for releasing all EGFR ligands, including epithelial AREG. AREG activates stromal EGFR, elicits reciprocal response to orchestrate mammary epithelial development [219], and acts as a paracrine regulator of E2-induce ductal morphogenesis [209,220]. AREG is also present during post-pubertal mammary development. AREG transcripts are substantially enriched in TEBs, and ducts’ growth compared to the epithelium-free stroma [209,219]. Thus, ADAM17–EGFR axis acts as an essential paracrine pathway in mammary gland development.

AREG activity downstream of the EGFR family triggers intracellular Ras/mitogen-activated protein kinase (MAPK) and PI3-K/Akt signaling pathways for its mitogenic action [221]. The branching or morphogenetic activity of MAPK is downstream of TNFα and EGFR, whereas transient proliferation action of MAPK is downstream of FGF7 and FGFR2 [104]. The mammary may use the temporal responses to integrate and interpret distinct signals [74]. Several other receptor tyrosine kinases have profound effects on pubertal mammary development, including Ron receptor tyrosine kinase (RON, also known as MSPR) [222], ephrin type-A receptor 2 [223], and fibroblast growth factor receptors (FGFRs) [209].

#### 3.5.5. Hormonal Regulation of Transcription Factors

Various hormones and growth factors regulate gene expression through receptors ligand-activated transcription factors and converting the hormonal and growth factors stimulus into a transcription response. Multiple transcription factors are involved in the process of mammary ductal morphogenesis, including AP-2γ (TFAP2G/TFAP2C), Gata-3, E-cadherin repressors Snail, Slug, and Twist, six families of homeodomain transcription factors, Sim2, CCAAT/enhancer-binding protein, C/EBPβ, Lef1, and DNA-binding protein inhibitor Id1 [101,103,224,225]. AP-2gamma (AP-2γ) is expressed in the cap cell layer and a subset of body cells of TEBs and regulates genetic processes downstream of ovarian hormones [225]. Gata-3 is a GATA family member and contains two GATA-type zinc fingers that regulate T cell development supports endothelial cells and ductal outgrowth [226,227]. Gata-3 binds directly to the promoter of the forkhead transcription factor FOXA1, which is required to bind ERa to chromatin and supports E2 signaling in the mammary gland [228]. FOXA1 mediates crosstalk between GATA3 and ERα signaling [4].

The E-cadherin repressors Snail and Slug, and the basic helix–loop–helix transcription factor Twist, are critical regulators of mammary gland development. A significant upregulation of the Snail and Twist transcripts in TEBs compared to mature ducts suggests that EMT regulators may regulate epithelial plasticity in the mammary gland [227]. Snail and Slug also regulate tight junction stability [229], gap junctional protein expression [230], desmosome disassembly [231], and protease expressions. Indeed, Snail and Slug are expressed in response to numerous EMT stimuli, including FGF and Wnt signaling [232,233], Lbx1 [234], TGF-β signaling [235], loss of Sim2 expression [236], and hypoxia [237]. Similarly, Twist represses E-cadherin, induces mesenchymal gene expression, and initiates invasion [238]. Like Snail and Slug, Twist targets numerous EMT-inducing stimuli, including hypoxia and Msx2 [239,240]. The convergence of multiple EMT pathways on Snail, Slug, and Twist are critical nodes in the networking of EMT signaling.

The Six families of homeodomain transcription factors, mainly, Six1, Six2, and Six4, regulate EMT. Six1 is commonly expressed in the embryonic, neonatal, and pubertal mammary glands; thereafter, its expression significantly decreased [241]. However, the functions of the Six family proteins in mammary gland development remain unclear.

Sim2 is a basic helix loop helix/Per-Amt-Sim (bHLH/PAS) transcription factor repressor. Sim2 is involved in mammary gland development by regulating epithelial plasticity, duct hollowing, and apicobasal polarity [236,242,243].

The CCAAT/enhancer-binding protein, C/EBPβ, has a critical function in promoting proper lobuloalveolar development in the mammary gland [151,244]. These factors are necessary for the proper development of ductal trees in the virgin mammary gland and subsequent development during pregnancy [245].

#### 3.5.6. Matrix Metalloproteinases (MMPs)

Matrix remodeling is required for cells to sprout from the main ducts and form branches for endbud progression [246]. In addition to GF receptors, MMPs as epithelial and stromal proteases are expressed differentially in mammary tissues as a local regulator of mammary branching by creating a path for TEBs in the surrounding ECM and fat pad [247,248]. MMP2 (gelatinase A) expresses in the epithelia near lateral branching and promotes cell survival. In contrast, MMP3 (stromelysin 1) expresses throughout the stroma and induces the local degradation of BM collagen IV and laminin at the site of lateral branching [249,250]. Interestingly, MMP1 is present in both stroma and epithelial cells [251]. MMP2 cleaves laminin-332 (LN332) fragments from the γ2 chain, which binds to the EGFR [252]. LN332 is an essential fragment in the mammary gland during mammary morphogenesis, which promotes proliferation by activating integrin signaling directly [252,253,254].

MMP9 expresses homogeneously with low levels in both the epithelium and the stroma [250]. MMP9 has an inhibitory role in pubertal mammary development [255]. MMP14 expresses in and around the TEBs [250,251]. An inhibitor of MMP14, the tissue inhibitor of metalloproteinases 3 (TIMP3), is downregulated in and around the TEBs; whereas TIMP1 does not inhibit MMP14 being upregulated at these sites [209]. There is a crosstalk between MMP14 and GFs during mammary development through cell motility. A high level of MMP14 signaling through the CD44 surface receptor and the RHO pathway promotes motility and directional persistence of migrating cells [256].

#### 3.5.7. Transforming Growth Factor β (TGFβ)

Tissue geometry and side branches are regulated through morphogenesis created by the autocrine–paracrine signaling morphogen and coordinated events of ECM turnover [198,257,258]. TGFβ is a ubiquitously expressed cytokine and determines the mammary gland’s tissue geometry as a morphogen [259]. TGFβ (TGFβ1, TGFβ2, and TGFβ3) is secreted within epithelium as a large latent complex (LLC) in all the stages of the mammary development. TGFβ is associated with fibrillar ECM proteins deposition and inhibition of proliferation [260]. TGFβ1 and TGFβ3 have an overlapping expression pattern in the epithelium during ductal elongation [261,262]. TGFβ3 is only expressed within cap cell layers of TEBs. TGFβ2 expresses very low levels in TEBs and is upregulated during pregnancy. TGFβ is activated by proteolytic cleavage of the latency-associated peptide (LAP) through the conformational change in LAP through binding to the αvβ6 integrin receptor or by MMP-9 thrombospondin or plasmin [263,264]. TGFβ activity is dependent on fibrillar ECM proteolysis or altered matrix stiffness. The mechanical tension is also involved in the release of TGFβ from the ECM [265,266]. The secreted TGFβ binds to target cells through TGFβ receptor type I/II and initiates multiple signaling cascades, including the canonical Smad signaling pathway [267], promoting branch initiation and maintaining proper ductal spacing within the duct-ensheathing ECM [149,267]. During puberty, high levels of E2 inhibit TGFβ expression, and low levels of TGFβ stimulate branching morphogenesis, whereas high levels of TGFβ inhibit ductal growth [268,269,270]. Importantly, Wnt5a is an essential mediator of TGFβ, suggesting that low thresholds of β-catenin signaling are maintained during pubertal ductal morphogenesis through TGFβ and Wnt5a antagonism. Highly localized expression of TGFβ in the TEBs control bifurcation by increasing ECM deposition within the cleft [261,269]. TGFβ provides additional support for mechanical tension regulating ECM function through fibroblast traction force that leads to large-scale directional patterning of fibrillar ECM proteins, including collagen I [271].

#### 3.5.8. Axonal Guidance Molecules

Several other signaling molecules are expressed during mammary gland development and support tissues morphogenesis and TEBs elongation and branching similar to axonal growth and migrations. These molecules include SLIT2 and its receptor ROBO, Netrin1/Neogenin, brain acid-soluble protein 1 (BASP), small proline-rich protein 1A (sprr1A), and semaphoring 3B, which are expressed differentially in cap cells of TEBs [272,273,274].

### 3.6. Pattern Formation during Pubertal Mammary Morphogenesis

Mammary morphogenesis is regulated through epithelial–mesenchymal transition (EMT), and mesenchymal–epithelial transition (MET) generates mechanical stresses by individual cells [74,275]. EMT and MET transmit and concentrate into stress gradients spanning many cell lengths and ultimately affect the cell behavior (proliferation and apoptosis) pattern and state (differentiation) near to the future branch sites from quiescent ducts [74]. Studies using microfabrication-based culture models combined with computational approaches have demonstrated that within epithelial cells, endogenous mechanical stress gradient arises owing to contraction of the actin cytoskeleton by myosin motors [276,277] that transmit between adjacent cells in epithelial tissues through cadherin-mediated adhesions and lead to regulate mammary branching [74]. This mechanotransduction in mammary epithelial tissues senses mechanical stress through integrins and other mechanosensory proteins, including focal adhesion kinase (FAK) [278,279,280], and activates specific ductal branch sites [74,281,282]. The migration of individual epithelial/stromal cells follows an orchestrated choreography through actin-rich protrusions, and cell–ECM adhesions promote forward propulsion like amoeba movement [283,284]. EMT is used to increase cells’ collective motility during this morphogenesis while maintaining their connectivity [285]. A wide range of other extracellular signals induces cell motility and polarity, including GFs, chemokines, and ECM proteins along with spatially localized activation of intracellular signaling components including PI3K, MAPKS, SRC, and Rho GTPases [74,286,287]. Moreover, many signaling pathways involved in the regulation of EMT regulation are also present in the TEB, including Wnt, FGF, TGFb, slug, and snail.

A genome-wide transcript analysis has shown that the EMT-related transcription factors SNAI1 (SNAIL1), TWIST1, and TWIST2 are expressed in the TEB microenvironment [227]. Advances in real-time imaging have identified large-scale coordinated movements of epithelial cells as a critical aspect of pubertal mammary development through cellular migration. Time-lapse confocal imagings of primary organoids have shown that the advanced TEBs consist of multi-layered luminal epithelial cells that rearrange dynamically and exhibit reduced apicobasal polarity [74,288]. Ultimately, the molecular symphony in the EMT through ECM drives mammary morphogenesis.

The patterning of the mammary gland is characterized by long and thin ducts with branch spacing and depending on the periodic location of progenitor cells. The progenitors are maintained within a stem cell niche, which depends on integrin–matrix interactions, and controlled by lateral inhibition signals, including notch and planar cell polarity. These intracellular forces are provided by localized cadherin expression and cytoskeleton contraction with cell expansion in the ducts longitudinally [28,29,289,290]. Branching morphogenesis is a complex process regulated by various factors expressed in the epithelium and stroma, including hormones and growth factors, ECM, MMPs, morphogens, and immune cells [6]. These molecules ultimately involve stromal–epithelial interaction and provide positional and orientation cues for ductal morphogenesis as collagen fibers radiate out from the TEB. Ductal development follows topographical cues in the ECM [259,291]. Ultimately, an orchestrated interactive complex network of intra- and inter-cellular microenvironment in the epithelial, luminal, and basal cells and the stroma develop mammary gland. In women, the mammary epithelial ductal structures proliferate and undergo increased branching during the luteal phase and then regress during the follicular phase [292].

## 4. Anatomy of Adult Mammary Gland

A non-lactating mammary gland comprises glandular (secretory) and adipose (fatty) tissue with fibrous connective tissue called Cooper’s ligaments. The glandular tissue has 15 to 20 lobes and lobules containing 10 to 100 alveoli (0.12 mm in diameter) [293]. The lobe size varies from 20 to 30 folds [294]. The 15–25 ducts drain the alveoli and merge into larger ducts that eventually converge into the central milk duct, which dilates slightly to form the laticiferous sinus before narrowing as nit passes through the nipple and open into the nipple surface. The nipple has different 5–9 ducts on average [295]. The diameter of the main ducts in the non-lactating mammary gland is 1.2–2.5 mm. The nipple pores are 0.4–0.7 mm in diameter and are surrounded by circular muscle fibers [11,296]. The glandular and adipose tissue ratio is 1:1 on average and declines with advancing age [297] (Figure 1, Figure 3 and Figure 4).

The blood supply in the mammary gland is by the anterior and posterior medial branches of the internal mammary artery (60%) and the lateral mammary branch of the lateral thoracic artery (30%) [298]. The posterior intercostal and pectoral branches of the thoracoacromial arteries are the sources for smaller blood supply [296]. The central venous drainage of the mammary gland is by deep and superficial systems connected with short veins, which drain into the internal thoracic, axillary, and cephalic veins. The corresponding mammary arteries follow the deep veins. The superficial plexus consists of subareolar veins from the nipple that drain into the periareolar vein, circle the nipples, and connect the superficial and deep plexus. Seventy-five percent of lymphatic drainage is by the axillary nodes, and the rest of the drainage from the deep portion of the mammary gland is by the internal mammary nodes [296]. The second to sixth intercostal nerves supply the mammary gland. Anterior nerves support subcutaneous tissues, whereas nipple and areola are supplied by the anterior and lateral cutaneous branches of the third and fifth intercostal nerves [299].

### 4.1. Differentiation of Mammary Gland during Pregnancy

The onset of pregnancy, progesterone (P4) and prolactin (PRL), and other systemic factors complete the morphologic maturation of the mammary gland. The tertiary branching and alveolar growth with differentiation through tissue remodeling accommodate the needs of the expanding epithelium [8,150,300,301,302,303,304,305]. During pregnancy, mammary branching occurs through two distinct phases: an early or proliferative phase and the second secretory or differentiation (functional) phase. The proliferative phase starts with the onset of conception (early pregnancy), characterized by rapid and extensive proliferation of mammary epithelial cells within the ductal branches, developing alveoli, and enhanced survival [59,306]. This process promotes the mammary epithelial cells population with thousand folds increase in cytokeratin and claudin [307,308]. The significant increase in the mammary gland is usually completed by week 22 of pregnancy. However, some considerable growth happened during the third trimester or postpartum. Mammary gland growth is directly correlated with human placental lactogen (hPL) concentrations during pregnancy. The second phase starts at the mid-pregnancy (differentiation phase) with the progression of ductules to secretory acini formation. The proliferation of the epithelium is reduced to a minimum with the beginning of the accumulation of lipids, stromal remodeling with enhancing angiogenesis, infiltration of macrophages and granulocytes, and fibroblast reorganization. The TDLUs further develop into milk-producing secretory lobular alveoli to support in accumulation of colostrum [309]. Ultimately, the lobules increase in number and size at the end of pregnancy, and adipocytes are slowly replaced by lobuloalveolar structures, leaving a scant amount of stroma. Thus, alveologenesis transform the virgin mammary gland into a 7–10 times heavier lactating mammary gland [72,143,310]. Before parturition, the cell proliferation decreases from the peak proliferative stage to a quiescent stage [311].

During pregnancy, mammary blood flow approximately doubles in volume. This increased blood flow promotes metabolic activity and temperature of the mammary gland. The elevated blood flow persists during lactation and decline to pre-pregnancy levels about the second week after weaning [312]. During pregnancy, the areola darkens in color, and the Montgomery glands, a combination of sebaceous and mammary milk glands, increase in size. The secretion of these glands provides maternal protection from both the mechanical stress of sucking and pathogenic invasion and a means of communication with the infants via odor.

Based on cell lineage, alveoli are formed from alveolar precursors (APs) during pregnancy, which gives rise to basal and luminal cells that differentiate into milk-producing cells [313]. The remodeling process supports the generation of new blood vessels, infiltration of immune and inflammatory cells, fibroblast reorganization, and the loss of lipid droplets within adipocytes to supply nutrients to the lobulo-alveoli for synthesizing and secreting milk during lactation [314,315,316,317,318,319]. This dramatic expansion of the alveolar epithelium at the end of ductule clusters forms acini (alveoli) by increasing tertiary branches through the invasion of the mammary fat pad. The formation of milk-secreting acinar units termed alveologenesis helps rapid outgrowth of the mammary gland during pregnancy. With the progression of pregnancy, the size and number of lobules increases to 20–40. These lobules are called terminal duct lobular units (TDLUs). TDLUs together form 15–20 major lobes in a pregnant woman’s breast [309] (Table 3, Figure 3)**.** Ultimately, a fully differentiated mammary gland has ramified branched ducts with epithelial trees composed of milk-secreting alveoli embedded in the fatty stroma. The inner layer of the lumen in ducts and alveoli is composed of a single layer of secretory epithelial cells and joined by the luminal surface by tight junctional complexes that produce milk components, whereas a basement membrane surrounds the outer layer of the basal myoepithelial cell, which supports contractile force for milk expulsion. Women who have never given birth to a viable or live infant are called Virginal or Nulliparous. The virginal woman has alveolar buds cluster around a terminal duct and is surrounded by bilayer epithelium.

### 4.2. Regulators of Mammary Development during Pregna

During pregnancy, hormonal requirements for mammary gland development are entirely different from pubertal ductal development. E2 regulates ductal development during puberty, whereas P4 and prolactin (PRL) are the main contributors to tubuloalveolar development during pregnancy [150,153,300,301,302,305]. “E2 prime” mammary epithelium is essential for the action of P4. Once side branches are established at mid-pregnancy, further alveologenesis requires epithelial PRL signaling [319]. The sequential action ensures that the distinct morphological steps occur orderly to find adequate space to unfold all the ducts before alveoli bud formation and transport secretions efficiently [161]. The ECM modulates all hormonal (E2, P4, and PRL) signals in MECs [304,320,321] (Figure 3 and Figure 4).

#### 4.2.1. Progesterone (P4) and Progesterone Receptor (PR)

In women, P4 is produced by the corpus luteum (CL) after ovulation and by the placenta after the eighth week of pregnancy. In mice, P4 is produced by CL throughout the pregnancy. P4 acts as a mitogen for mammary epithelial cells and induces extensive side-branching and alveologenesis [301,302,322]. Intracellular action of P4 takes place through progesterone receptors (PRa and PRβ) and regulates the expression of PR target genes [323,324,325]. The PR expression in the luminal mammary epithelium is required for epithelial cell proliferation but not for the stroma [326,327]. The ratio of PRa: PRβ varies in humans (1:1) and in mice (2:1–3:1). PRβ is expressed in the mammary gland’s epithelial and stromal compartments [258,301]. PR specifies spatial patterns of alveolar progenitors by inducing β-catenin signaling and regulation of cellular transcriptional activity [328,329]. The P promotes the proliferation of neighboring cells and supports the maturation of alveoli synergistically through multiple paracrine factors, including IGF-1, E2, receptor activator of nuclear factor κβ (NF-κβ)-ligand (RANK-L), transcription factors, mainly, signal transducer and activator of transcription-5a, STAT5A, and STAT5B, prolactin receptor (PRLR), ErbB4, Wnt, etc. [330,331,332,333,334]. The differential regulation of β-catenin transcription activity is by the critical kinases of NF-κB signaling pathways (IκB kinase IKKα and IKKβ) [335]. IKKα phosphorylation increases the stability and transcriptional activity of β-catenin, while phosphorylation by IKKβ decreases both [335]. Interestingly, P through PR signaling regulates β-catenin responsiveness in the ducts but not at the ductal tips [4]. During pregnancy, Wnts and RANKL also function downstream of P in a paracrine to stimulate side-branching and alveolar development through luminal cells [330,331,333]. Early to mid-pregnancy, multiple Wnt RNAs, namely, Wnt4, Wnt5b, and Wnt6, peak sequentially, and are directly elevated by P [336,337]. Ectopic Wnt1 and Wnt4 expression acts as a local mediator for P action and promotes side branching [326,338], and is able to rescue impaired ductal side-branching [28,330,336,337]. Thus, Wnts and RANKL promote stem cell amplification and branching by paracrine induction of β-catenin signaling in basal cells. In contrast, β-catenin signaling in luminal progenitors is essential for alveologenesis and induced by Wnt-independent mechanisms [33]. P also regulates FN and other ECM protein expression, including Areg in the mammary gland [170,219].

#### 4.2.2. Prolactin (PRL) and Prolactin Receptor (PRLR)

PRL is a peptide hormone synthesized and secreted by pituitary lactotrophs, supports the corpus luteum (CL) maintenance during early pregnancy, and induces mammary development [339]. PRL ensures the secretion of E2 and P from the CL and placenta, which require tubuloalveolar proliferation and functional differentiation [340]. After mid-pregnancy, both functions of PRL are replaced by placental lactogens (PL), but PRL takes over its role again after birth. PRL is essential for alveologenesis and differentiation of MEC into milk-producing cells during late pregnancy. At parturition, the fall in circulating P, in the context of elevated PRL, triggers the switch to secretory lactation, where tight junctions between epithelia close and colostrum accumulate in the alveolar lumen. PRL with P promotes the differentiation of the alveoli to secrete milk during lactation [341].

Intracellular action of PRL is through PRL-receptor (PRLR) threshold levels. PRLR is a transmembrane protein of the class I cytokine receptor superfamily member couple with nonreceptor tyrosine kinases [305,331]. PRLR is phosphorylated by Janus kinase-2 (JAK2) at specific tyrosine residue upon PRL binding with PRLR dimerization and conformational changes, which activates STAT5 [342,343,344], mitogen-activated protein kinase (MAPK)-signaling, ELF5 [304,345], GATA-3 [226], SOCS [346], insulin-like growth factor-2 (IGF-2) [319], and galanin [94]. STAT5a is the principal STAT5 isoform in PRL action on the mammary gland and transcription of milk protein genes during pregnancy and lactation [177,319]. The eight members of the SOCS family (SOCS1-7 and cytokine-inducible Src-homology, SH2; protein, CIS) interact with JAKs and cytokine receptors to curtail the activation of STAT protein [347]. SOCS1 is linked to the modulation of interferon signaling in the immune system and essential regulator of PRL signaling in mammary epithelium during pregnancy through STAT5 activity [348].

PRL also activates multiple proliferative and differentiation factors, including Caveolin-1 (CAV-1) RANK-L. CAV-1 is a structural component of lipid rafts (caveolae) and is required for controlling concomitant precocious STAT5 activity during pregnancy [349]. RANK-L is required for later steps of alveolar differentiation through downstream activation of NF-κβ [350]. Both the RANK-NF-κβ signaling pathway and STAT5 activate the cyclin D1 gene and promote alveolar proliferation. STAT5 activates cyclin D1 through a g-IFN activated site (GAS) within the promoter [351,352]. Genes for milk protein, including acidic whey protein (WAP) and β-casein, are activated by STAT5 through the GAS promoter sequence [353,354]. STAT5 activates multiple survival pathway genes through GAS motif promoters, including Bcl2 family proteins [355]. Studies have shown that STAT6 and its upstream cytokines IL4 and IL13 are essential for expanding the luminal lineage [356]. STAT6 phosphorylation is directly correlated with the expression of IL4Ra and GATA3 in the epithelium, followed by c-MAF induction later in pregnancy [4]. In the absence of STAT6, a 70% decrease in the number of alveoli at day 5 of gestation diminishes epithelial cell proliferation.

PRL has a cross talk with integrins through PRLR signaling in luminal cells of alveoli. Integrins act as micro-environmental checkpoints and sustained PRL signals to support epithelial cells to correct spatial location within the tissues [357,358,359,360].

PRL signaling activates ELF5, a member of the ETS family of a transcription factor in mammary epithelial cells, to support lobuloalveolar development and alveolar differentiation [94,308,361,362]. The expression of ELF5 is promoted by STAT5 and regulates the milk protein gene WAP during pregnancy but not lactation [345].

#### 4.2.3. Cortisol

Cortisol plays an essential physiological role in mammary gland development in the latter part of pregnancy and during lactation. At this stage, placental lactogens stimulate DNA synthesis in the mammary cells, and cortisol induces the formation of the rough endoplasmic reticulum, where milk proteins will be synthesized [363]. Cortisol also sensitizes mammary tissues to modulate during stress and impact E2 activity [364]. Cortisol responds to stress signals through the cytoplasmic glucocorticoid receptor (GR) and promotes protein, lipid, and carbohydrate catabolism and immune functions [365,366]. Interestingly, PRL release upon birth causes lobular differentiation and the secretion of early milk proteins (β-casein). In contrast, cortisol predominantly regulates the expression of late milk proteins and regulates β-casein expression [367]. GR activity is important for the ductal development of the virgin mammary gland [368], lobuloalveolar development during pregnancy [369], and in stimulating milk production during lactation. However, cortisol misregulation and prolonged presence during periods of stress expose breast cells to activate downstream biological pathways outside their normal context [364].

#### 4.2.4. ErbB Receptors

During late pregnancy, alveolar differentiation and maintenance are supported by ErbB4 [52]. Studies have shown that STAT5 is activated by tyrosine phosphorylation when it is closely associated with ErbB4 [52,370]. EGFR and ErbB2 are most prominently involved in ductal morphogenesis during puberty, alveolar morphogenesis in late pregnancy, and support lactation. In contrast, ErbB-3 and ErbB-4 contribute most during pregnancy and lactation in response to NRGs [177]. PRL signaling engages the ErbB4/HER4 pathway via JAK2, and the ErbB4 provides an important component of STAT5a-dependent lactogenic differentiation [371].

#### 4.2.5. Inhibitor of Differentiation-1 (Id-1)

Id-1 supports the proliferation of lobuloalveolar structures during early and mid-pregnancy. Id-1 supports invasion, migration, and anti-apoptosis for mammary epithelial cells [103]. Id-1 regulates the expression of Wnt/β-catenin, phospho-Akt, BMP2, FGF3, and RAR-β in tubuloalveolar development in mammary glands [103].

#### 4.2.6. Activin

Activin and its receptors are regulators of branching morphogenesis during pregnancy and support lactation [372,373]. In pregnant women, circulating total Activin A concentrations are 100 times greater than serum concentrations in cycling women [374]. Interestingly, both Activin A and follistatin are present in human breast milk from the beginning of the first day to throughout the lactation period [375]. Activin B expression is highest from mid to late lactation and decreases during involution. During lactation, there is also consistent TGFβ dependent nuclear expression and localization of Smad3. Activin subunits are differentially expressed in the nulliparous and pregnant mammary glands [376]. Activin subunit β supports ductal elongation and alveolar development [313]. Interestingly, loss of activin signaling results in lactation failure, while loss of TGFβ signaling allows alveogenesis and production of β-casein [377]. Studies suggest that activin βB is required for ductal elongation, branching, alveogenesis, and lactation.

#### 4.2.7. Extracellular Matrix (ECM)

In adult women, the non-lactating mammary glands have 80% or more stroma, whereas, during pregnancy, a high level of ECM expressions and secretions. Among ECM proteins, tenascins (TN) expression levels increase ~200-fold in pregnancy and lactation and rapidly decline with involution [378]. The TN family has five members (TN-C, TN-R, TN-W, TN-X, and TN-Y) with molecular weight 180–300 kDa. TN family proteins provide anti-adhesive properties to ECM via disrupting the interactions among TM heparan sulfate receptor syndecan-4, a5β1 integrin receptor, and fibronectin that support stretch force in epithelial cells [379]. The conditional deletion of the β1-integrin gene leads to reduced mammary outgrowth and a failure of lactation that coincides with a loss of transcriptional factor STAT5 activity [127,380]. The deletion of dystroglycan in mammary cells has inhibited STAT5 activation and milk-protein expression, whereas dystroglycan knockout results in defective gland outgrowth, lactation, and STAT5 activity. Integrins are the key signaling receptors in the matrix for regulation of STAT5 activity, whereas downstream, they require the adaptor and integrin-linked kinases and Rac, but not focal adhesion kinase (Fak) [381,382]. β1-integrins, but not β4-integrins, specifically regulate PRL signaling. Other studies have shown that deletion of a3- or a6-integrins does not cause a lactation defect like losing the entire β1-integrin family [126].

The ECM protein, biglycan transcript, is abundant in the mammary gland and increases four- to five-fold during the transition from lactation to involution [383,384]. Elastic fibers are crucial in the lactating mammary gland as the tensile forces continuously change due to suckling, milk accumulation during nursing, and myoepithelial contraction on the secretory alveolar epithelial cells [81]. Mice lacking fibronectin (Fn) showed lobuloalveolar impairment from lobular hypoplasia to aplasia [101]. Mammary epithelial cells in Fn-knockout mice have a slower proliferation rate with concomitantly decreased expression of integrin β1 (Itgβ1) and lack of autophosphorylation of Fak. Ultimately, it fails to undergo normal lobuloalveolar differentiation during pregnancy [101].

#### 4.2.8. Protein Kinase Cδ, Multiple B-Cell Leukemia/Lymphoma 2 (Bcl-2), and Ductal Lumen Formation

The PKCδ protein is expressed at all stages of mammary development. However, there are limited studies on PKCδ and normal mammary gland development. PKCδ is a ubiquitously expressed isoform of the PKC family of serine/threonine kinases and regulator of epithelial cell apoptosis through its localization, tyrosine phosphorylation, and the presence of other pro- and antiapoptotic signaling molecules. PKCδ plays a key role during mammary development at puberty [224]. PKCδ expression is highest in TEBs during mid-pregnancy when apoptosis in the highest-level results in luminal hollowing and during involution [5,66,385]. Apoptosis within the body cells of the TEB is critical for ductal morphogenesis of the mammary gland [66]. The lumen is formed behind the endbud as the duct develops from the nipple to the extremities of its branches. The apoptosis of these cells can be reduced by the overexpression of the pro-survival BCL2 factor. There are wide range of prosurvival proteins are expressed during branching morphogenesis, including multiple B-cell leukemia/lymphoma 2 (Bcl-2) family members. Bcl2 family is known to play a pivotal role in the regulation of apoptosis as checkpoints between the cell surface and internal death signals, the formation of the apoptosome, and the activation of the caspase cascade. Several Bcl2 family members are highly expressed in the mammary gland, including antiapoptotic B-cell leukemia/lymphoma X (Bcl-x), Bcl-2 and B-cell leukemia/lymphoma w (Bcl-w), as well as the proapoptotic Bcl-2-associated X protein (Bax) and Bcl-2 homologous antagonist/killer (Bak) proteins. However, over-expression of the Bcl-2 gene in mice caused abnormal ductal development, whereas mice in which the proapoptotic protein, Bcl-2-interacting mediator of death (Bim), is deleted show delayed apoptosis in the TEBs, supporting a role for apoptosis during ductal morphogenesis. Studies using null mice for *Bim* (*Bcl2l11*), a BH3-only-domain regulator of apoptosis has revealed that BIM is essential for removing the surplus epithelium in the duct [386]. In the absence of BIM-mediated cell death, these cells switch to a more squamous cell type and die via a caspase-3-independent mechanism. Bim also detects loss of GF signals rather than altered integrin signaling [387]. Ductal lumen formation is regulated apoptosis by altering GF delivery to the endbud. Thus, Bim helps in cavitation and thereby creates the lumen [386]. In contrast, apoptosis and proliferation in the TEBs are unaffected in PKCδ-/- mice and reduced ductal branching [388].

The role of cell–matrix interactions in the formation of alveolar lumens during lactation is not established. Therefore, lumens may form in the mammary gland by fluid movement rather than apoptosis as in other epithelial cells [389]. In this process, apical-basal polarity may provide the key mechanism for lumen formation. The apical surface of luminal epithelia is decorated with transmembrane mucins (MUC-1), which are heavily glycosylated and prevent cell adhesion at surfaces. In three-dimensional cultures of mammary and MDCK cells, LM-111, dystroglycan, and β1-integrins have central roles in establishing polarity [234,384,390]. Thus, ECM provides a guiding role within the alveolar epithelium to orient luminal cells and create luminal surfaces and thus, fluid-filled cavities [384]. However, the exact intracellular mechanisms in controlling the formation of the lumen have remained unknown.

The mechanical environment, including matrix stiffness, governs mammary epithelial functional differentiation and supports milk protein synthesis in soft environments but not stiff environments [391]. In this process, ECM plays an essential role through mechanical stress-regulated transcription and by altering the branching through the regulation of MMPs [392,393]. During pregnancy, the cells within alveoli undergo complete differentiation, polarize, and form the secretory alveoli capable of producing milk proteins and cytoplasmic lipid droplets. The reduction of P4 in the context of the continued presence of PRL at parturition activates the secretory function of these cells characterized by the movement of milk proteins and fat globules (lipid droplets) into the lumen [304,394].

## 5. The Lactating Mammary Glands

In lactating mammary glands, there is a huge expansion of the vasculature within the stroma to support milk production through supplying a large number of various micro-and macro-molecules [315]. A diffuse layer of contractile basal-myoepithelial cell surrounds the secretory epithelial cells in the alveoli, whereas the smooth muscles surround the areola-nipple complex. Myoepithelial cells support the collecting and ejection of milk from the 15 to 20 lobular ducts, which are connected to interlobular ducts. In turn, each lobe drains milk into the nipple. The myoepithelial cells contract in response to oxytocin stimulation, which results in milk release, whereas the high levels of P inhibit milk production during pregnancy [12]. The mammary glands of a non-pregnant woman have few alveoli that are budded off from the end of the ducts.

## 6. Stromal Regulation of Involution

The mammary gland remodeling is critical for removing the epithelial or milk-secreting alveolar epithelial cells after menstruation or weaning or if pregnancy does not occur (from day 27 to menstruation). Upon weaning or the cessation of suckling, more than ninety percent of epithelial cells in the differentiated alveoli undergo apoptosis and are removed by macrophages and a subset of surviving mammary epithelial cells [395,396]. Apoptosis of epithelial cells proceeds with the disappearance of the stromal edema and overall regression in the size of the lobules that remodeled tissue architecture before the future reproductive period [184,397]. Thus, the mammary gland undergoes progressive remodeling back to a virgin-like state through a collectively called “involution” process. This is the dramatic deconstruction of the epithelial cell of the lactating mammary gland and restoring/remodeling of stroma to a pre-pregnancy/virgin gland [4] (Figure 4).

There are three phases of involution. The first involution stage is an initial reversible phase due to milk accumulation locally within an alveolar lumen. The levels of systemic lactogenic hormones fall with an increase in caspase activity [185]; although this process can be restored by milk producing cells within few hours of milk accumulation, and lactation phase can be re-established by suckling within 48 h. In this phase, individual mammary epithelial cells die by apoptosis with shedding, alveolar cell death, along with breakdown of tight junctions, infiltration of leukocytes and phagocytosis. This process is regulated locally by milk stasis, but the general structure of the mammary gland is maintained. The first phase apoptosis is inhibited by the STAT5a and interferon regulatory factor-1 (IRF-1) and promoted by STAT3 and TGFβ3 [327,398].

The second involution phase is characterized by apoptosis through proteinases from lysosomes and mediated by lactogenic hormones. In this phase, the action of MMPs and caspases together removes connective tissues and BMs. During early mammary gland involution, high levels of TIMPs inhibit MMPs activity. In contrast, the second phase of high involution levels of MMPs (MMP-2,-3,-11), serine proteases, and urokinase-type plasminogen activator (uPA), and low levels of TIMPs enhance extensive remodeling of the ECM and stromal components of the mammary gland. The Zinc transporter zinc transporter 2 (ZnT2) is also involved in this involution process. Ultimately, the epithelial cells lose their adhesion to a basement membrane, which is destroyed by the increased proteinase activity [185]. As a result, the cells lose survival signals from the ECM, redifferentiate fat cells, and finally destroy the ductal tree. Many apoptotic cells and debris are removed by phagocytosis, mainly by macrophages. E2 and P regulate ductal involution along with multiple autocrine-paracrine factors, including milk components, namely, lactalbumin, LIF-STAT3-axis, TGFβ3-axis, and death receptor-axis, and integrin adhesion receptors that stimulate apoptosis in the ductal tree [185,399,400]. STAT3 and STAT5 are reciprocally activated in the mammary gland. STAT5 promotes survival and milk secretion, while STAT3 stimulates apoptosis. LIF activates STAT3 [401,402], whereas STAT3 induces transcription of IGFBP-5, interleukin-6 (IL6), and C/ebpδ [185,403]. During the second phase of involution, the absence of Smad3 in mammary epithelium promotes TGFβ-mediated apoptosis [404]. Other studies have shown that SOCS3 binds to a tyrosine residue in the GP130 receptor, a shared subunit of receptors for cytokines such as interleukin-6 (IL-6) and leukemia inhibitory factor (LIF) during involution of the mammary gland [385,401,405,406,407,408]. The irreversible phase of involution is supported by the recruitment of macrophages, immune and other phagocytic cells that removes the cellular debris. The third phase is a biosynthesis phase, and stroma is remodeled and repopulated with adipogenic cells or tissues, filling the space of the regressed epithelium.

## 7. Mammary Gland at Single Cell Resolution

In recent years, our understanding of the complex signaling and tissue remodeling during mammary gland development, puberty, pregnancy, lactation, and involution are broadened by the revolutionary spatial transcriptomics and single-cell RNA sequencing (scRNA seq) technologies in a combination of fuorescence-activated cell sorting or laser-capture microdissection of a specific cell population or tissues. Single-cell RNA-seq is a developing technology that achieves the refinement of gene expression analysis from the bulk of cells deep into the single-cell level.

The differentiation of mammary epithelial cells was analyzed using single-cell transcriptomic in murine and human mammary epithelial cells [409,410,411,412,413]. These studies suggested that a typical luminal progenitor cell in the virgin mammary gland gives rise to hormone-responsive mature luminal cells, whereas during pregnancy, these cells give rise to secretory alveolar cells [409,410,411,412,413]. Interestingly, the post-parous mammary gland contained primed parity-induced luminal progenitor cells that upregulated lactation-associated genes. Moreover, human primary mammary epithelial cells isolated from milk compared to resting, non-lactating breast tissue using single-cell RNA sequencing suggest that human milk largely contains epithelial cells belonging to the luminal lineage and immune cells [413]. These recent studies provide a brief reference map and a window on the cellular dynamics that occur during mammary development during puberty, pregnancy, and lactation.

## 8. Conclusions

The development and functional differentiation of the mammary gland is a complex morphogenetic process. Unlike most epithelial organs, mammary glands develop postnatally and undergo complex epithelial remodeling throughout puberty, pregnancy, lactation, and weaning. The multiple stages of proliferation and differentiation at puberty and lactation are mainly regulated by concurrent alterations in key hormones and growth factors across various reproductive states and stages. Mouse model, cell-culture, 3D-culture system, knock-out models, and organoids helped us to elucidate the embryonic mammary development and programming of epithelial tissue into a complex organ through EMT. Epithelial cells influence the growth and differentiation of the tissues that surround them and vice versa through ECM. During pubertal and lactation, EMT and epithelial–mesenchymal interaction are critical for the growth and development of the mammary gland in a 3D space, and that ultimately regulates the physiological and metabolic state of the mammary gland. Epithelial apoptosis has a key role in the development and functioning of the mammary gland, with the formation of ducts during puberty and the removal of excess epithelial cells after lactation. Using the genetically modified mouse models, the development of stem cell enrichment procedures, the culture embryonic mammary glands, and three-dimensional culture models have provided new insight into the developmental secrets of the mammary gland. However, our identification of novel signaling pathways is a very early stage in understanding lineage commitment and tubulogenesis regulation.

## Figures and Tables

**Table 1:** Classification of human embryonic mammary gland development [4,5,10,11,12,13].

Stage	Embryo or Fetus Size	Gestational Stage (in Week, W)
Ridge stage	<5 mm embryo	4 W, around day 35
Milk hill stage	>5.5 mm embryo	4–6 W
Mammary disc stage	~10–11 mm embryo	7–8 W
Lobule type stage	11–25 mm embryo	8–9 W
Cone stage	25–30 mm embryo	9–10 W
Budding stage	30–68 mm embryo	11–12 W
Identification stage	68 mm–10 cm embryo	12–15 W
Branching stage	10 cm fetus	15–20 W
Canalization stage	28 cm fetus	22–32 W
End vesicle/Newborn stage (Monolayer of epithelium and contain colostrum)		6 months

**Table 2:** Classification of human mammary gland development based on Tanner Scale.

Stage	Age	Characteristics
Tanner I	10 y or younger	Glandular tissues absent, areola follows the skin contour of chest
Tanner II	10–11.5 y	Mammary bud forms with small area of surrounding glandular tissue and areola begins to widen
Tanner III	11.5–13 y	Mammary begins to more elevated and extends beyond the borders of the areola, which continues to widen but remains in contour with surrounding breast
Tanner IV	13–15 y	Increased mammary size and elevation, areola and papilla form a secondary mound projecting from the contour of the surrounding mammary gland
Tanner V	15 y or above	Mammary reaches final adult size, areola returns to contour of the surrounding mammary with a projecting central papilla

**Table 3:** Parity status of women mammary gland.

Type	Women Stage	Characteristics
I	Virginal or Nulliparous (never given birth to a viable or live infant)	Alveolar bud cluster around a terminal duct, and terminal ducts/alveolar buds are surrounded by bilayer epithelium (~11 ductules)
II	Parous (given birth to a viable or live infant)	Ductules increases in number (~47 ductules), sprouting of new alveolar buds
III	Parous	Ductules increases in number (~80 ductules)
IV	Lactating	Maximal functional differentiation and development, milk secreting lobules

## Data Availability

Not applicable.

## References

[1] Hovey R.C., Trott J.F., Vonderhaar B.K. (2002). Establishing a framework for the functional mammary gland: From endocrinology to morphology. J. Mammary Gland Biol. Neoplasia.

[2] Peaker M. (2002). The mammary gland in mammalian evolution: A brief commentary on some of the concepts. J. Mammary Gland Biol. Neoplasia.

[3] Muschler J., Streuli C.H. (2010). Cell-matrix interactions in mammary gland development and breast cancer. Cold Spring Harb. Perspect. Biol..

[4] Watson C.J., Khaled W.T. (2008). Mammary development in the embryo and adult: A journey of morphogenesis and commitment. Development.

[5] Sternlicht M.D. (2006). Key stages in mammary gland development: The cues that regulate ductal branching morphogenesis. Breast Cancer Res..

[6] Visvader J.E. (2009). Keeping a breast of the mammary epithelial hierarchy and breast tumorigenesis. Genes Dev..

[7] Streuli C.H. (2003). Cell adhesion in mammary gland biology and neoplasia. J. Mammary Gland Biol. Neoplasia.

[8] Cowin P., Wysolmerski J. (2010). Molecular mechanisms guiding embryonic mammary gland development. Cold Spring Harb. Perspect. Biol..

[9] Parmar H., Cunha G.R. (2004). Epithelial-stromal interactions in the mouse and human mammary gland in vivo. Endocr.-Relat. Cancer.

[10] Javed A., Lteif A. (2013). Development of the Human Breast. Semin. Plast. Surg..

[11] Vorherr H. (1974). The Breast: Morphology, Physiology and Lactation.

[12] Howard B.A., Gusterson B.A. (2000). Human breast development. J. Mammary Gland Biol. Neoplasia.

[13] Russo J., Russo I.H., Neville M.C., Daniel C.W. (1987). The Mammary Gland: Development, Regulation and Function.

[14] Jolicoeur F. (2005). Intrauterine breast development and the mammary myoepithelial lineage. J. Mammary Gland Biol. Neoplasia.

[15] Sakakura T., Neville M.C., Daniel C.W. (1987). Mammary embryogenesis. The Mammary Gland: Development, Regulation and Function.

[16] Hens J.R., Wysolmerski J.J. (2005). Key stages of mammary gland development: Molecular mechanisms involved in the formation of the embryonic mammary gland. Breast Cancer Res..

[17] Hinck L., Silberstein G.B. (2005). Key stages in mammary gland development: The mammary end bud as a motile organ. Breast Cancer Res..

[18] Chu E.Y., Hens J., Andl T., Kairo A., Yamaguchi T.P., Brisken C., Glick A., Wysolmerski J.J., Millar S.E. (2004). Canonical WNT signaling promotes mammary placode development and is essential for initiation of mammary gland morphogenesis. Development.

[19] Veltmaat J.M., Van Veelen W., Thiery J.P., Bellusci S. (2004). Identification of the mammary line in mouse by Wnt10b expression. Dev. Dyn..

[20] Foley J., Dann P., Hong J., Cosgrove J., Dreyer B., Rimm D., Dunbar M., Philbrick W., Wysolmerski J. (2001). Parathyroid hormone-related protein maintains mammary epithelial fate and triggers nipple skin differentiation during embryonic breast development. Development.

[21] Kratochwil K., Gwatkin R.B.L. (1987). Tissue combination and organ culture studies in the development of the embryonic mammary gland. Developmental Biology: A Comprehensive Synthesis.

[22] Masso-Welch P.A., Darcy K.M., Stangle-Castor N.C., Ip M.M. (2000). A developmental atlas of rat mammary gland histology. J. Mammary Gland Biol. Neoplasia.

[23] Hennighausen L., Robinson G.W. (2001). Signaling pathways in mammary gland development. Dev. Cell.

[24] Staal F.J., Chhatta A., Mikkers H. (2016). Caught in a Wnt storm: Complexities of Wnt signalling in hematopoiesis. Exp. Hematol..

[25] McNeill H., Woodgett J.R. (2010). When pathways collide: Collaboration and connivance among signalling proteins in development. Nat. Rev. Mol. Cell Biol..

[26] van Amerongen R., Nusse R. (2009). Towards an integrated view of Wnt signaling in development. Development.

[27] Komiya Y., Habas R. (2008). Wnt signal transduction pathways. Organogenesis.

[28] Brennan K.R., Brown A.M. (2004). Wnt proteins in mammary development and cancer. J. Mammary Gland Biol. Neoplasia.

[29] Bouras T., Pal B., Vaillant F., Harburg G., Asselin-Labat M.L., Oakes S.R., Lindeman G.J., Visvader J.E. (2008). Notch signaling regulates mammary stem cell function and luminal cell-fate commitment. Cell Stem Cell.

[30] Lindvall C., Evans N.C., Zylstra C.R., Li Y., Alexander C.M., Williams B.O. (2006). The Wnt signaling receptor Lrp5 is required for mammary ductal stem cell activity and Wnt1-induced tumorigenesis. J. Biol. Chem..

[31] Lindvall C., Zylstra C.R., Evans N., West R.A., Dykema K., Furge K.A., Williams B.O. (2009). The Wnt co-receptor Lrp6 is required for normal mouse mammary gland development. PLoS ONE.

[32] Eblaghie M.C., Song S.J., Kim J.Y., Akita K., Tickle C., Jung H.S. (2004). Interactions between FGF and Wnt signals and *Tbx3* gene expression in mammary gland initiation in mouse embryos. J. Anat..

[33] Incassati A., Chandramouli A., Eelkema R., Cowin P. (2010). Key signaling nodes in mammary gland development and cancer: β-catenin. Breast Cancer Res..

[34] Christiansen J.H., Dennis C.L., Wicking C.A., Monkley S.J., Wilkinson D.G., Wainwright B.J. (1995). Murine Wnt-11 and Wnt-12 have temporally and spatially restricted expression patterns during embryonic development. Mech. Dev..

[35] Hens J., Dann P., Hiremath M., Pan T.C., Chodosh L., Wysolmerski J. (2009). Analysis of gene expression in PTHrP^−/−^ mammary buds supports a role for BMP signaling and MMP2 in the initiation of ductal morphogenesis. Dev. Dyn..

[36] Hynes N.E., Watson C.J. (2010). Mammary gland growth factors: Roles in normal development and in cancer. Cold Spring Harb. Perspect. Biol..

[37] Pond A.C., Bin X., Batts T., Roarty K., Hilsenbeck S., Rosen J.M. (2013). Fibroblast growth factor receptor signaling is essential for normal mammary gland development and stem cell function. Stem Cells.

[38] Mailleux A.A., Spencer-Dene B., Dillon C., Ndiaye D., Savona-Baron C., Itoh N., Kato S., Dickson C., Thiery J.P., Bellusci S. (2002). Role of FGF10/FGFR2b signaling during mammary gland development in the mouse embryo. Development.

[39] Veltmaat J.M., Relaix F., Le L.T., Kratochwil K., Sala F.G., van Veelen W., Rice R., Spencer-Dene B., Mailleux A.A., Rice D.P. (2006). Gli3-mediated somitic Fgf10 expression gradients are required for the induction and patterning of mammary epithelium along the embryonic axes. Development.

[40] Walterhouse D.O., Lamm M.L., Villavicencio E., Iannaccone P.M. (2003). Emerging roles for hedgehog-patched-Gli signal transduction in reproduction. Biol. Reprod..

[41] Dai P., Akimaru H., Tanaka Y., Maekawa T., Nakafuku M., Ishii S. (1999). Sonic Hedgehog-induced activation of the Gli1 promoter is mediated by GLI3. J. Biol. Chem..

[42] Ikram M.S., Neill G.W., Regl G., Eichberger T., Frischauf A.M., Aberger F., Quinn A., Philpott M. (2004). GLI2 is expressed in normal human epidermis and BCC and induces GLI1 expression by binding to its promoter. J. Investig. Dermatol..

[43] Hatsell S.J., Cowin P. (2006). Gli3-mediated repression of Hedgehog targets is required for normal mammary development. Development.

[44] Kleinberg D.L., Wood T.L., Furth P.A., Lee A.V. (2009). Growth hormone and insulin-like growth factor-I in the transition from normal mammary development to preneoplastic mammary lesions. Endocr. Rev..

[45] Heckman B.M., Chakravarty G., Vargo-Gogola T., Gonzales-Rimbau M., Hadsell D.L., Lee A.V., Settleman J., Rosen J.M. (2007). Crosstalk between the p190-B RhoGAP and IGF signaling pathways is required for embryonic mammary bud development. Dev. Biol..

[46] Hens J.R., Dann P., Zhang J.P., Harris S., Robinson G.W., Wysolmerski J. (2007). BMP4 and PTHrP interact to stimulate ductal outgrowth during embryonic mammary development and to inhibit hair follicle induction. Development.

[47] Wysolmerski J.J., Philbrick W.M., Dunbar M.E., Lanske B., Kronenberg H., Broadus A.E. (1998). Rescue of the parathyroid hormone-related protein knockout mouse demonstrates that parathyroid hormone-related protein is essential for mammary gland development. Development.

[48] Gritli-Linde A., Hallberg K., Harfe B.D., Reyahi A., Kannius-Janson M., Nilsson J., Cobourne M.T., Sharpe P.T., McMahon A.P., Linde A. (2007). Abnormal hair development and apparent follicular transformation to mammary gland in the absence of hedgehog signaling. Dev. Cell.

[49] Howard B., Panchal H., McCarthy A., Ashworth A. (2005). Identification of the scaramanga gene implicates Neuregulin3 in mammary gland specification. Genes Dev..

[50] Hardy K.M., Booth B.W., Hendrix M.J., Salomon D.S., Strizzi L. (2010). ErbB/EGF signaling and EMT in mammary development and breast cancer. J. Mammary Gland Biol. Neoplasia.

[51] Wansbury O., Panchal H., James M., Parry S., Ashworth A., Howard B. (2008). Dynamic expression of Erbb pathway members during early mammary gland morphogenesis. J. Investig. Dermatol..

[52] Tidcombe H., Jackson-Fisher A., Mathers K., Stern D.F., Gassmann M., Golding J.P. (2003). Neural and mammary gland defects in ErbB4 knockout mice genetically rescued from embryonic lethality. Proc. Natl. Acad. Sci. USA.

[53] Harris H.A., Albert L.M., Leathurby Y., Malamas M.S., Mewshaw R.E., Miller C.P., Kharode Y.P., Marzolf J., Komm B.S., Winneker R.C. (2003). Evaluation of an estrogen receptor-β agonist in animal models of human disease. Endocrinology.

[54] Davenport T.G., Jerome-Majewska L.A., Papaioannou V.E. (2003). Mammary gland, limb and yolk sac defects in mice lacking Tbx3, the gene mutated in human ulnar mammary syndrome. Development.

[55] Bamshad M., Lin R.C., Law D.J., Watkins W.C., Krakowiak P.A., Moore M.E., Franceschini P., Lala R., Holmes L.B., Gebuhr T.C. (1997). Mutations in human TBX3 alter limb, apocrine and genital development in ulnar-mammary syndrome. Nat. Genet..

[56] Phippard D.J., Weber-Hall S.J., Sharpe P.T., Naylor M.S., Jayatalake H., Maas R., Woo I., Roberts-Clark D., Francis-West P.H., Liu Y.H. (1996). Regulation of Msx-1, Msx-2, Bmp-2 and Bmp-4 during foetal and postnatal mammary gland development. Development.

[57] Satokata I., Ma L., Ohshima H., Bei M., Woo I., Nishizawa K., Maeda T., Takano Y., Uchiyama M., Heaney S. (2000). Msx2 deficiency in mice causes pleiotropic defects in bone growth and ectodermal organ formation. Nat. Genet..

[58] Dunbar M.E., Dann P.R., Robinson G.W., Hennighausen L., Zhang J.P., Wysolmerski J.J. (1999). Parathyroid hormone-related protein signaling is necessary for sexual dimorphism during embryonic mammary development. Development.

[59] Russo J., Russo I.H. (2004). Development of the human breast. Maturitas.

[60] Capuco A.V., Ellis S.E. (2013). Comparative aspects of mammary gland development and homeostasis. Annu. Rev. Anim. Biosci..

[61] Michael Akers R. (2017). A 100-Year Review: Mammary development and lactation. J. Dairy Sci..

[62] Williams J.M., Daniel C.W. (1983). Mammary ductal elongation: Differentiation of myoepithelium and basal lamina during branching morphogenesis. Dev. Biol..

[63] Daniel C.W., Smith G.H. (1999). The mammary gland: A model for development. J. Mammary Gland Biol. Neoplasia.

[64] Bai L., Rohrschneider L.R. (2010). s-SHIP promoter expression marks activated stem cells in developing mouse mammary tissue. Genes Dev..

[65] Rios A.C., Fu N.Y., Lindeman G.L., Visvader J.E. (2014). In situ identification of bipotent stem cells in the mammary gland. Nature.

[66] Humphreys R.C., Krajewska M., Krnacik S., Jaeger R., Weiher H., Krajewski S., Reed J.C., Rosen J.M. (1996). Apoptosis in the terminal endbud of the murine mammary gland: A mechanism of ductal morphogenesis. Development.

[67] Ewald A.J., Huebner R.J., Palsdottir H., Lee J.K., Perez M.J., Jorgens D.M., Tauscher A.N., Cheung K.J.C.-Z., Auer M. (2012). Mammary collective cell migration involves transient loss of epithelial features and individual cell migration within the epithelium. J. Cell Sci..

[68] Paine I., Chauviere A., Landua J., Sreekumar A., Cristini V., Rosen J., Lewis M.T. (2016). A geometrically-constrained mathematical model of mammary gland ductal elongation reveals novel cellular dynamics within the terminal end Bud. PLoS Comput. Biol..

[69] Fata J.E., Chaudhary V., Khokha R. (2001). Cellular turnover in the mammary gland is correlated with systemic levels of progesterone and not 17β-estradiol during the estrous cycle. Biol. Reprod..

[70] Ferguson J.E., Schor A.M., Howell A., Ferguson M.W. (1992). Changes in the extracellular matrix of the normal human breast during the menstrual cycle. Cell Tissue Res..

[71] Potten C.S., Watson R.J., Williams G.T., Ticle S., Roberts S.A., Harris M., Howell A. (1988). The effect of age and the menstrual cycle upon proliferative activity of the normal human breast. Br. J. Cancer.

[72] Lester S.C. (2005). Differential diagnosis of granulomatous mastitis. Breast J..

[73] Metcalfe A.D., Gilmore A., Klinowska T., Oliver J., Valentijn A.J., Brown R., Ross A., MacGregor G., Hickman J.A., Streuli C.H. (1999). Developmental regulation of Bcl-2 family protein expression in the involuting mammary gland. J. Cell Sci..

[74] Gjorevski N., Nelson C.M. (2011). Integrated morphodynamic signalling of the mammary gland. Nat. Rev. Mol. Cell Biol..

[75] McNally S., Martin F. (2011). Molecular regulators of pubertal mammary gland development. Ann. Med..

[76] Butcher D.T., Alliston T., Weaver V.M. (2009). A tense situation: Forcing tumour progression. Nat. Rev. Cancer.

[77] Fata J.E., Werb Z., Bissell M.J. (2004). Regulation of mammary gland branching morphogenesis by the extracellular matrix and its remodeling enzymes. Breast Cancer Res..

[78] Ekblom P., Lonai P., Talts J.F. (2003). Expression and biological role of laminin-1. Matrix Biol..

[79] Jalkanen M., Rapraeger A., Bernfield M. (1988). Mouse mammary epithelial cells produce basement membrane and cell surface heparan sulfate proteoglycans containing distinct core proteins. J. Cell Biol..

[80] Kalluri R. (2003). Basement membranes: Structure, assembly and role in tumour angiogenesis. Nat. Rev. Cancer.

[81] Maller O., Martinson H., Schedin P. (2010). Extracellular matrix composition reveals complex and dynamic stromal-epithelial interactions in the mammary gland. J. Mammary Gland Biol. Neoplasia.

[82] Guilak F., Cohen D.M., Estes B.T., Gimble J.M., Liedtke W., Chen C.S. (2009). Control of stem cell fate by physical interactions with the extracellular matrix. Cell Stem Cell.

[83] Yurchenco P.D., Patton B.L. (2009). Developmental and pathogenic mechanisms of basement membrane assembly. Curr. Pharm. Des..

[84] Tzu J., Marinkovich M.P. (2008). Bridging structure with function: Structural, regulatory, and developmental role of laminins. Int. J. Biochem. Cell Biol..

[85] Aumailley M., Bruckner-Tuderman L., Carter W.G., Deutzmann R., Edgar D., Ekblom P., Engel J., Engvall E., Hohenester E., Jones J.C. (2005). A simplified laminin nomenclature. Matrix Biol..

[86] Prince J.M., Klinowska T.C., Marshman E., Lowe E.T., Mayer U., Miner J., Aberdam D., Vestweber D., Gusterson B., Streuli C.H. (2002). Cell-matrix interactions during development and apoptosis of the mouse mammary gland in vivo. Dev. Dyn..

[87] Delehedde M., Lyon M., Sergeant N., Rahmoune H., Fernig D.G. (2001). Poteoglycans: Pericellular and cell surface multireceptors that integrate external stimuli in the mammary gland. J. Mammary Gland Biol. Neoplasia.

[88] Barresi R., Campbell K.P. (2006). Dystroglycan: From biosynthesis to pathogenesis of human disease. J. Cell Sci..

[89] Morgan M.R., Humphries M.J., Bass M.D. (2007). Synergist.tic control of cell adhesion by integrins and syndecans. Nat. Rev. Mol. Cell Biol..

[90] Vogel W.F., Aszodi A., Alves F., Pawson T. (2001). Discoidin domain receptor 1 tyrosine kinase has an essential role in mammary gland development. Mol. Cell Biol..

[91] Midwood K.S., Valenick L.V., Hsia H.C., Schwarzbauer J.E. (2004). Coregulation of fibronectin signaling and matrix contraction by tenascin-C and syndecan-4. Mol. Biol. Cell..

[92] Shattil S.J., Kim C., Ginsberg M.H. (2010). The final steps of integrin activation: The end game. Nat. Rev. Mol. Cell Biol..

[93] Takada Y., Ye X., Simon S. (2007). The integrins. Genome Biol..

[94] Naylor M.J., Ginsburg E., Iismaa T.P., Vonderhaar B.K., Wynick D., Ormandy C.J. (2003). The neuropeptide galanin augments lobuloalveolar development. J. Biol. Chem..

[95] Friedrich M.V., Gohring W., Morgelin M., Brancaccio A., David G., Timpl R. (1999). Structural basis of glycosaminoglycan modification and of heterotypic interactions of perlecan domain V. J. Mol. Biol..

[96] Schedin P., Mitrenga T., McDaniel S., Kaeck M. (2004). Mammary ECM composition and function are altered by reproductive state. Mol. Carcinog..

[97] Gouon-Evans V., Rothenberg M.E., Pollard J.W. (2000). Postnatal mammary gland development requires macrophages and eosinophils. Development.

[98] Ingman W.V., Wyckoff J., Gouon-Evans V., Condeelis J., Pollard J.W. (2006). Macrophages promote collagen fibrillogenesis around terminal end buds of the developing mammary gland. Dev. Dyn..

[99] Unsworth A., Anderson R., Britt K. (2014). Stromal fibroblasts and the immune microenvironment: Partners in mammary gland biology and pathology?. J. Mammary Gland Biol. Neoplasia.

[100] Zhang X., Martinez D., Koledova Z., Qiao G., Streuli C.H., Lu P. (2014). FGF ligands of the postnatal mammary stroma regulate distinct aspects of epithelial morphogenesis. Development.

[101] Liu J., Esmailpour T., Shang X., Gulsen G., Liu A., Huang T. (2011). TBX3 over-expression causes mammary gland hyperplasia and increases mammary stem-like cells in an inducible transgenic mouse model. BMC Dev. Biol..

[102] Sakai T., Larsen M., Yamada K.M. (2003). Fibronectin requirement in branching morphogenesis. Nature.

[103] Shin D.H., Jang S.H., Kang B.C., Kim H.J., Oh S.H., Kong G. (2011). Constitutive overexpression of Id-1 in mammary glands of transgenic mice results in precocious and increased formation of terminal end buds, enhanced alveologenesis, delayed involution. J. Cell Physiol..

[104] Fata J.E., Mori H., Ewald A.J., Zhang H., Yao E., Werb Z., Bissell M.J. (2007). The MAPK (ERK-1,2) pathway integrates distinct and antagonistic signals from TGFα and FGF7 in morphogenesis of mouse mammary epithelium. Dev. Biol..

[105] Barker T.H., Baneyx G., Cardó-Vila M., Workman G.A., Weaver M., Menon P.M., Dedhar S., Rempel S.A., Arap W., Pasqualini R. (2005). SPARC regulates extracellular matrix organization through its modulation of integrin-linked kinase activity. J. Biol. Chem..

[106] Wong S.Y., Crowley D., Bronson R.T., Hynes R.O. (2008). Analyses of the role of endogenous SPARC in mouse models of prostate and breast cancer. Clin. Exp. Metastasis.

[107] Schaefer L., Iozzo R.V. (2008). Biological functions of the small leucine-rich proteoglycans: From genetics to signal transduction. J. Biol. Chem..

[108] Minor K., Tang X., Kahrilas G., Archibald S.J., Davies J.E., Davies S.J. (2008). Decorin promotes robust axon growth on inhibitory CSPGs and myelin via a direct effect on neurons. Neurobiol. Dis..

[109] Wight T.N., Heinegard D.K., Hascall V.C., Hay E.D. (1991). Proteoglycans Structure and Function. Cell Biology of Extracellular Matrix.

[110] Zhu J.X., Goldoni S., Bix G., Owens R.T., McQuillan D.J., Reed C.C., Iozzo R.V. (2005). Decorin evokes protracted internalization and degradation of the epidermal growth factor receptor via caveolar endocytosis. J. Biol. Chem..

[111] Xu T., Bianco P., Fisher L.W., Longenecker G., Smith E., Goldstein S., Bonadio J., Boskey A., Heegaard A.M., Sommer B. (1998). Targeted disruption of the biglycan gene leads to an osteoporosis-like phenotype in mice. Nat. Genet..

[112] Schaefer L., Mihalik D., Babelova A., Krzyzankova M., Gröne H.J., Iozzo R.V., Young M.F., Seidler D.G., Lin G., Reinhardt D.P. (2004). Regulation of fibrillin-1 by biglycan and decorin is important for tissue preservation in the kidney during pressure-induced injury. Am. J. Pathol..

[113] Mecham R.P., Heuser J.E., Hay E.D. (1991). Cell Biology of Extracellular Matrix.

[114] Reinboth B., Hanssen E., Cleary E.G., Gibson M.A. (2002). Molecular interactions of biglycan and decorin with elastic fiber components: Biglycan forms a ternary complex with tropoelastin and microfibril-associated glycoprotein 1. J. Biol. Chem..

[115] Trask B.C., Trask T.M., Broekelmann T., Mecham R.P. (2000). The microfibrillar proteins MAGP-1 and fibrillin-1 form a ternary complex with the chondroitin sulfate proteoglycan decorin. Mol. Biol. Cell.

[116] Silberstein G.B., Daniel C.W. (1982). Glycosaminoglycans in the basal lamina and extracellular matrix of the developing mouse mammary duct. Dev. Biol..

[117] Discher D.E., Mooney D.J., Zandstra P.W. (2009). Growth factors, matrices, and forces combine and control stem cells. Science.

[118] Hynes R.O. (2009). The extracellular matrix: Not just pretty fibrils. Science.

[119] Hall A. (2009). The cytoskeleton and cancer. Cancer Metastasis Rev..

[120] Pollard T.D., Cooper J.A. (2009). Actin, a central player in cell shape and movement. Science.

[121] Caswell P.T., Vadrevu S., Norman J.C. (2009). Integrins: Masters and slaves of endocytic transport. Nat. Rev. Mol. Cell Biol..

[122] Levental K.R., Yu H., Kass L., Lakins J.N., Egeblad M., Erler J.T., Fong S.F., Csiszar K., Giaccia A., Weninger W. (2009). Matrix crosslinking forces tumor progression by enhancing integrin signaling. Cell.

[123] Lorand L., Graham R.M. (2003). Transglutaminases: Crosslinking enzymes with pleiotropic functions. Nat. Rev. Mol. Cell Biol..

[124] Pullan S., Wilson J., Metcalfe A., Edwards G.M., Goberdhan N., Tilly J., Hickman J.A., Dive C., Streuli C.H. (1996). Requirement of basement membrane for the suppression of programmed cell death in mammary epithelium. J. Cell Sci..

[125] Runswick S.K., O’Hare M.J., Jones L., Streuli C.H., Garrod D.R. (2001). Desmosomal adhesion regulates epithelial morphogenesis and cell positioning. Nat. Cell Biol..

[126] Klinowska T.C., Soriano J.V., Edwards G.M., Oliver J.M., Valentijn A.J., Montesano R., Streuli C.H. (1999). Laminin and β1 integrins are crucial for normal mammary gland development in the mouse. Dev. Biol..

[127] Naylor M.J., Li N., Cheung J., Lowe E.T., Lambert E., Marlow R., Wang P., Schatzmann F., Wintermantel T., Schüetz G. (2005). Ablation of β1 integrin in mammary epithelium reveals a key role for integrin in glandular morphogenesis and differentiation. J. Cell Biol..

[128] Gehler S., Baldassarre M., Lad Y., Leight J.L., Wozniak M.A., Riching K.M., Eliceiri K.W., Weaver V.M., Calderwood D.A., Keely P.J. (2009). Filamin A-β1 integrin complex tunes epithelial cell response to matrix tension. Mol. Biol. Cell.

[129] Glukhova M.A., Streuli C.H. (2013). How integrins control breast biology. Curr. Opin. Cell Biol..

[130] Taddei I., Deugnier M.A., Faraldo M.M., Petit V., Bouvard D., Medina D., Fässler R., Thiery J.P., Glukhova M.A. (2008). β1 integrin deletion from the basal compartment of the mammary epithelium affects stem cells. Nat. Cell Biol..

[131] Rebustini I.T., Patel V.N., Stewart J.S., Layvey A., Georges-Labouesse E., Miner J.H., Hoffman M.P. (2007). Laminin α5 is necessary for submandibular gland epithelial morphogenesis and influences FGFR expression through β1 integrin signaling. Dev. Biol..

[132] Iozzo R.V. (2005). Basement membrane proteoglycans: From cellar to ceiling. Nat. Rev. Mol. Cell Biol..

[133] Liu Y., Chattopadhyay N., Qin S., Szekeres C., Vasylyeva T., Mahoney Z.X., Taglienti M., Bates C.M., Chapman H.A., Miner J.H. (2009). Coordinate integrin and c-Met signaling regulate Wnt gene expression during epithelial morphogenesis. Development.

[134] Castets M., Coissieux M.M., Delloye-Bourgeois C., Bernard L., Delcros J.G., Bernet A., Laudet V., Mehlen P. (2009). Inhibition of endothelial cell apoptosis by netrin-1 during angiogenesis. Dev. Cell.

[135] Hagedorn E.J., Yashiro H., Ziel J.W., Ihara S., Wang Z., Sherwood D.R. (2009). Integrin acts upstream of netrin signaling to regulate formation of the anchor cell’s invasive membrane in *C. elegans*. Dev. Cell.

[136] Strizzi L., Postovit L.M., Margaryan N.V., Seftor E.A., Abbott D.E., Seftor R.E., Salomon D.S., Hendrix M.J. (2008). Emerging roles of nodal and Cripto-1: From embryogenesis to breast cancer progression. Breast Dis..

[137] Couldrey C., Moitra J., Vinson C., Anver M., Nagashima K., Green J. (2002). Adipose tissue: A vital in vivo role in mammary gland development but not differentiation. Dev. Dyn..

[138] Kamikawa A., Ichii O., Yamaji D., Imao T., Suzuki C., Okamatsu-Ogura Y., Terao A., Kon Y., Kimura K. (2009). Diet-induced obesity disrupts ductal development in the mammary glands of nonpregnant mice. Dev. Dyn..

[139] Landskroner-Eiger S., Park J., Israel D., Pollard J.W., Scherer P.E. (2010). Morphogenesis of the developing mammary gland: Stage-dependent impact of adipocytes. Dev. Biol..

[140] Kamalati T., Niranjan B., Yant J., Buluwela L. (1999). HGF/SF in mammary epithelial growth and morphogenesis: In vitro and in vivo models. J. Mammary Gland Biol. Neoplasia.

[141] Kleinberg D.L., Feldman M., Ruan W. (2000). IGF-I: An essential factor in terminal end bud formation and ductal morphogenesis. J. Mammary Gland Biol. Neoplasia.

[142] Knox S.M., Lombaert I.M., Reed X., Vitale-Cross L., Gutkind J.S., Hoffman M.P. (2010). Parasympathetic innervation maintains epithelial progenitor cells during salivary organogenesis. Science.

[143] Atabai K., Sheppard D., Werb Z. (2007). Roles of the innate immune system in mammary gland remodeling during involution. J. Mammary Gland Biol. Neoplasia.

[144] Lilla J.N., Werb Z. (2010). Mast cells contribute to the stromal microenvironment in mammary gland branching morphogenesis. Dev. Biol..

[145] Russell J.S., McGee S.O., Ip M.M., Kuhlmann D., Masso-Welch P.A. (2007). Conjugated linoleic acid induces mast cell recruitment during mouse mammary gland stromal remodeling. J. Nutr..

[146] Aupperlee M.D., Zhao Y., Tan Y.S., Leipprandt J.R., Bennett J., Haslam S.Z., Schwartz R.C. (2014). Epidermal growth factor receptor (EGFR) signaling is a key mediator of hormone-induced leukocyte infiltration in the pubertal female mammary gland. Endocrinology.

[147] Horiuchi T., Weller P.F. (1997). Expression of vascular endothelial growth factor by human eosinophils: Upregulation by granulocyte macrophage colony-stimulating factor and interleukin-5. Am. J. Respir Cell Mol. Biol..

[148] Gusterson B.A., Warburton M.J., Mitchell D., Ellison M., Munro Neville A., Rudland P.S. (1982). Distribution of Myoeithelial Cells and Basement Membrane Proteins in the Normal Breast and in Benign and Malignant Breast Diseases. Cancer Res..

[149] Silberstein G.B. (2001). Postnatal mammary gland morphogenesis. Microsc. Res. Tech..

[150] Bocchinfuso W.P., Lindzey J.K., Hewitt S.C., Clark J.A., Myers P.H., Cooper R., Korach K.S. (2000). Induction of mammary gland development in estrogen receptor-α knockout mice. Endocrinology.

[151] Grimm S.L., Rosen J.M. (2003). The role of C/EBPβ in mammary gland development and breast cancer. J. Mammary Gland Biol. Neoplasia.

[152] Kleinberg D.L., Ruan W. (2008). IGF-I, GH, and sex steroid effects in normal mammary gland development. J. Mammary Gland Biol. Neoplasia.

[153] Rusidz M., Adlanmrini M., Chantalat E., Letron I.R., Cayre S., Arnal J.F., Deugnier M.A., Lenfant F. (2021). Estrogen receptor-α signaling in postnatal mammary development and breast cancers. Cell. Mol. Life Sci..

[154] Fisher C.R., Graves K.H., Parlow A.F., Simpson E.R. (1998). Characterization of mice deficient in aromatase (ArKO) because of targeted disruption of the cyp19 gene. Proc. Natl. Acad. Sci. USA.

[155] Bocchinfuso W.P., Korach K.S. (1997). Mammary gland development and tumorigenesis in estrogen receptor knockout mice. J. Mammary Gland Biol. Neoplasia.

[156] Cheng G., Weihua Z., Warner M., Gustafsson J.A. (2004). Estrogen receptors ERα and ERβ in proliferation in the rodent mammary gland. Proc. Natl. Acad. Sci. USA.

[157] Curtis Hewitt S., Couse J.F., Korach K.S. (2000). Estrogen receptor transcription and transactivation: Estrogen receptor knockout mice: What their phenotypes reveal about mechanisms of estrogen action. Breast Cancer Res..

[158] Mallepell S., Krust A., Chambon P., Brisken C. (2006). Paracrine signaling through the epithelial estrogen receptor α is required for proliferation and morphogenesis in the mammary gland. Proc. Natl. Acad. Sci. USA.

[159] Feng Y., Manka D., Wagner K.U., Khan S.A. (2007). Estrogen receptor-α expression in the mammary epithelium is required for ductal and alveolar morphogenesis in mice. Proc. Natl. Acad. Sci. USA.

[160] Acconcia F., Kumar R. (2006). Signaling regulation of genomic and nongenomic functions of estrogen receptors. Cancer Lett..

[161] Brisken C., O’Malley B. (2010). Hormone action in the mammary gland. Cold Spring Harb. Perspect. Biol..

[162] Castoria G., Migliaccio A., Bilancio A., Di Domenico M., de Falco A., Lombardi M., Fiorentino R., Varricchio L., Barone M.V., Auricchio F. (2001). PI3-kinase in concert with Src promotes the S-phase entry of oestradiol-stimulated MCF-7 cells. EMBO J..

[163] Lombardi M., Castoria G., Migliaccio A., Barone M.V., Di Stasio R., Ciociola A., Bottero D., Yamaguchi H., Appella E., Auricchio F. (2008). Hormone-dependent nuclear export of estradiol receptor and DNA synthesis in breast cancer cells. J. Cell Biol..

[164] Migliaccio A., Di Domenico M., Castoria G., de Falco A., Bontempo P., Nola E., Auricchio F. (1996). Tyrosine kinase/p21ras/MAP-kinase pathway activation by estradiol-receptor complex in MCF-7 cells. EMBO J..

[165] Simoncini T., Hafezi-Moghadam A., Brazil D.P., Ley K., Chin W.W., Liao J.K. (2000). Interaction of oestrogen receptor with the regulatory subunit of phosphatidylinositol-3-OH kinase. Nature.

[166] Ciarloni L., Mallepell S., Brisken C. (2007). Amphiregulin is an essential mediator of estrogen receptor α function in mammary gland development. Proc. Natl. Acad. Sci. USA.

[167] Kenney N.J., Bowman A., Korach K.S., Barrett J.C., Salomon D.S. (2003). Effect of exogenous epidermal-like growth factors on mammary gland development and differentiation in the estrogen receptor-α knockout (ERKO) mouse. Breast Cancer Res. Treat..

[168] Sternlicht M.D., Sunnarborg S.W. (2008). The ADAM17-amphiregulin-EGFR axis in mammary development and cancer. J. Mammary Gland Biol. Neoplasia.

[169] Lu P., Ewald A.J., Martin G.R., Werb Z. (2008). Genetic mosaic analysis reveals FGF receptor 2 function in terminal end buds during mammary gland branching morphogenesis. Dev. Biol..

[170] Woodward T.L., Mienaltowski A.S., Modi R.R., Bennett J.M., Haslam S.Z. (2001). Fibronectin and the α_5_β_1_ integrin are under developmental and ovarian steroid regulation in the normal mouse mammary gland. Endocrinology.

[171] Liang Y., Brekken R.A., Hyder S.M. (2006). Vascular endothelial growth factor induces proliferation of breast cancer cells and inhibits the anti-proliferative activity of anti-hormones. Endocr. Relat. Cancer.

[172] Dabrosin C., Margetts P.J., Gauldie J. (2003). Estradiol increases extracellular levels of vascular endothelial growth factor in vivo in murine mammary cancer. Int. J. Cancer.

[173] Feldman M., Ruan W., Cunningham B.C., Wells J.A., Kleinberg D.L. (1993). Evidence that the growth hormone receptor mediates differentiation and development of the mammary gland. Endocrinology.

[174] Argetsinger L.S., Campbell G.S., Yang X., Witthuhn B.A., Silvennoinen O., Ihle J.N., Carter-Su C. (1993). Identification of JAK2 as a growth hormone receptor-associated tyrosine kinase. Cell.

[175] Zhang Y., Jiang J., Kopchick J.J., Frank S.J. (1999). Disulfide linkage of growth hormone (GH) receptors (GHR) reflects GH-induced GHR dimerization. Association of JAK2 with the GHR is enhanced by receptor dimerization. J. Biol. Chem..

[176] Carter-Su C., Schwartz J., Smit L.S. (1996). Molecular mechanism of growth hormone action. Annu. Rev. Physiol..

[177] Frank S.J. (2008). Mechanistic aspects of crosstalk between GH and PRL and ErbB receptor family signaling. J. Mammary Gland Biol. Neoplasia.

[178] Leung D.W., Spencer S.A., Cachianes G., Hammonds R.G., Collins C., Henzel W.J., Barnard R., Waters M.J., Wood W.I. (1987). Growth hormone receptor and serum binding protein: Purification, cloning and expression. Nature.

[179] Zeps N., Bentel J.M., Papadimitriou J.M., Antuono M.F.D., Dawkins H.J. (1998). Estrogen receptor-negative epithelial cells in mouse mammary gland development and growth. Differentiation.

[180] Macias H., Hinck L. (2012). Mammary gland development. Wiley Interdiscip. Rev. Dev. Biol..

[181] Cullen K.J., Allison A., Martire I., Ellis M., Singer C. (1992). Insulin-like growth factor expression in breast cancer epithelium and stroma. Breast Cancer Res. Treat..

[182] Cullen K.J., Smith H.S., Hill S., Rosen N., Lippman M.E. (1991). Growth factor messenger RNA expression by human breast fibroblasts from benign and malignant lesions. Cancer Res..

[183] Paik S. (1992). Expression of IGF-I and IGF-II mRNA in breast tissue. Breast Cancer Res. Treat..

[184] Gallego M.I., Binart N., Robinson G.W., Okagaki R., Coschigano K.T., Perry J., Kopchick J.J., Oka T., Kelly P.A., Hennighausen L. (2001). Prolactin, growth hormone, and epidermal growth factor activate Stat5 in different compartments of mammary tissue and exert different and overlapping developmental effects. Dev. Biol..

[185] Green K.A., Streuli C.H. (2004). Apoptosis regulation in the mammary gland. Cell. Mol. Life Sci..

[186] Corbit K.C., Aanstad P., Singla V., Norman A.R., Stainier D.Y., Reiter J.F. (2005). Vertebrate Smoothened functions at the primary cilium. Nature.

[187] Milenkovic L., Scott M.P., Rohatgi R. (2009). Lateral transport of Smoothened from the plasma membrane to the membrane of the cilium. J. Cell Biol..

[188] Rohatgi R., Milenkovic L., Scott M.P. (2007). Patched1 regulates hedgehog signaling at the primary cilium. Science.

[189] Hooper J.E., Scott M.P. (2005). Communicating with Hedgehogs. Nat. Rev. Mol. Cell Biol..

[190] Huangfu D., Liu A., Rakeman A.S., Murcia N.S., Niswander L., Anderson K.V. (2003). Hedgehog signalling in the mouse requires intraflagellar transport proteins. Nature.

[191] Varjosalo M., Taipale J. (2007). Hedgehog signaling. J. Cell Sci..

[192] Kogerman P., Grimm T., Kogerman L., Krause D., Undén A.B., Sandstedt B., Toftgård R., Zaphiropoulos P.G. (1999). Mammalian suppressor-of-fused modulates nuclear-cytoplasmic shuttling of Gli-1. Nat. Cell Biol..

[193] Bühler T.A., Dale T.C., Kieback C., Humphreys R.C., Rosen J.M. (1993). Localization and quantification of Wnt-2 gene expression in mouse mammary development. Dev. Biol..

[194] Lane T.F., Leder P. (1997). Wnt-10b directs hypermorphic development and transformation in mammary glands of male and female mice. Oncogene.

[195] Roarty K., Shore A.N., Creighton C.J., Rosen J.M. (2015). Ror2 regulates branching, differentiation, and actin-cytoskeletal dynamics within the mammary epithelium. J. Cell Biol..

[196] Neugebauer J.M., Amack J.D., Peterson A.G., Bisgrove B.W., Yost H.J. (2009). FGF signalling during embryo development regulates cilia length in diverse epithelia. Nature.

[197] Van Genderen C., Okamura R.M., Fariñas I., Quo R.G., Parslow T.G., Bruhn L., Grosschedl R. (1994). Development of several organs that require inductive epithelial-mesenchymal interactions is impaired in LEF-1-deficient mice. Genes Dev..

[198] Roarty K., Serra R. (2007). Wnt5a is required for proper mammary gland development and TGF-β-mediated inhibition of ductal growth. Development.

[199] Astigiano S., Damonte P., Barbieri O. (2003). Inhibition of ductal morphogenesis in the mammary gland of WAP-fgf4 transgenic mice. Anat. Embryol..

[200] Cui Y., Li Q. (2013). Expression and functions of fibroblast growth factor 10 in the mouse mammary gland. Int. J. Mol. Sci..

[201] Itoh N. (2016). FGF10: A multifunctional mesenchymal-epithelial signaling growth factor in development, health, and disease. Cytokine Growth Factor Rev..

[202] Parsa S., Ramasamy S.K., Langhe S.D., Gupte V.V., Haigh J.J., Medina D., Bellusci S. (2008). Terminal end bud maintenance in mammary gland is dependent upon FGFR2b signaling. Dev. Biol..

[203] Wiesen J.F., Young P., Werb Z., Cunha G.R. (1999). Signaling through the stromal epidermal growth factor receptor is necessary for mammary ductal development. Development.

[204] Schroeder J.A., Lee D.C. (1998). Dynamic expression and activation of ERBB receptors in the developing mouse mammary gland. Cell Growth Differ..

[205] Coleman S., Silberstein G.B., Daniel C.W. (1988). Ductal morphogenesis in the mouse mammary gland: Evidence supporting a role for epidermal growth factor. Dev. Biol..

[206] Sebastian J., Richards R.G., Walker M.P., Wiesen J.F., Werb Z., Derynck R., Hom Y.K., Cunha G.R., DiAugustine R.P. (1998). Activation and function of the epidermal growth factor receptor and erbB-2 during mammary gland morphogenesis. Cell Growth Differ..

[207] Xie W., Paterson A.J., Chin E., Nabell L.M., Kudlow J.E. (1997). Targeted expression of a dominant negative epidermal growth factor receptor in the mammary gland of transgenic mice inhibits pubertal mammary duct development. Mol. Endocrinol..

[208] Howlin J., McBryan J., Napoletano S., Lambe T., McArdle E., Shioda T., Martin F. (2006). CITED1 homozygous null mice display aberrant pubertal mammary ductal morphogenesis. Oncogene.

[209] Sternlicht M.D., Sunnarborg S.W., Kouros-Mehr H., Yu Y., Lee D.C., Werb Z. (2005). Mammary ductal morphogenesis requires paracrine activation of stromal EGFR via ADAM17-dependent shedding of epithelial amphiregulin. Development.

[210] Linggi B., Carpenter G. (2006). ErbB receptors: New insights on mechanisms and biology. Trends Cell Biol..

[211] Boeri Erba E., Bergatto E., Cabodi S., Silengo L., Tarone G., Defilippi P., Jensen O.N. (2005). Systematic analysis of the epidermal growth factor receptor by mass spectrometry reveals stimulation-dependent multisite phosphorylation. Mol. Cell. Proteomics.

[212] Guo L., Kozlosky C.J., Ericsson L.H., Daniel T.O., Cerretti D.P., Johnson R.S. (2003). Studies of ligand-induced site-specific phosphorylation of epidermal growth factor receptor. J. Am. Soc. Mass Spectrom..

[213] Wu S.L., Kim J., Bandle R.W., Liotta L., Petricoin E., Karger B.L. (2006). Dynamic profiling of the post-translational modifications and interaction partners of epidermal growth factor receptor signaling after stimulation by epidermal growth factor using Extended Range Proteomic Analysis (ERPA). Mol. Cell. Proteomics.

[214] Schulze W.X., Deng L., Mann M. (2005). Phosphotyrosine interactome of the ErbB-receptor kinase family. Mol. Syst. Biol..

[215] Jorissen R.N., Walker F., Pouliot N., Garrett T.P., Ward C.W., Burgess A.W. (2003). Epidermal growth factor receptor: Mechanisms of activation and signalling. Exp. Cell Res..

[216] Patel V.N., Knox S.M., Likar K.M., Lathrop C.A., Hossain R., Eftekhari S., Whitelock J.M., Elkin M., Vlodavsky I., Hoffman M.P. (2007). Heparanase cleavage of perlecan heparan sulfate modulates FGF10 activity during ex vivo submandibular gland branching morphogenesis. Development.

[217] Zcharia E., Metzger S., Chajek-Shaul T., Aingorn H., Elkin M., Friedmann Y., Weinstein T., Li J.P., Lindahl U., Vlodavsky I. (2004). Transgenic expression of mammalian heparanase uncovers physiological functions of heparan sulfate in tissue morphogenesis, vascularization, and feeding behavior. FASEB J..

[218] Luetteke N.C., Qiu T.H., Fenton S.E., Troyer K.L., Riedel R.F., Chang A., Lee D.C. (1999). Targeted inactivation of the EGF and amphiregulin genes reveals distinct roles for EGF receptor ligands in mouse mammary gland development. Development.

[219] Aupperlee M.D., Leipprandt J.R., Bennett J.M., Schwartz R.C., Haslam S.Z. (2013). Amphiregulin mediates progesterone-induced mammary ductal development during puberty. Breast Cancer Res..

[220] McBryan J., Howlin J., Napoletano S., Martin F. (2008). Amphiregulin: Role in mammary gland development and breast cancer. J. Mammary Gland Biol. Neoplasia.

[221] Kato M., Inazu T., Kawai Y., Masamura K., Yoshida M., Tanaka N., Miyamoto K., Miyamori I. (2003). Amphiregulin is a potent mitogen for the vascular smooth muscle cell line, A7r5. Biochem. Biophys. Res. Commun..

[222] Meyer S.E., Zinser G.M., Stuart W.D., Pathrose P., Waltz S.E. (2009). The Ron receptor tyrosine kinase negatively regulates mammary gland branching morphogenesis. Dev. Biol..

[223] Vaught D., Chen J., Brantley-Sieders D.M. (2009). Regulation of mammary gland branching morphogenesis by EphA2 receptor tyrosine kinase. Mol. Biol. Cell.

[224] Allen-Petersen B.L., Miller M.R., Neville M.C., Anderson S.M., Nakayama K.I., Reyland M.E. (2010). Loss of protein kinase C delta alters mammary gland development and apoptosis. Cell Death Dis..

[225] Jäger R., Schäfer S., Hau-Liersch M., Schorle H. (2010). Loss of transcription factor AP-2γ/TFAP2C impairs branching morphogenesis of the murine mammary gland. Dev. Dyn..

[226] Asselin-Labat M.L., Shackleton M., Stingl J., Vaillant F., Forrest N.C., Eaves C.J., Visvader J.E., Lindeman G.J. (2006). Steroid hormone receptor status of mouse mammary stem cells. J. Natl. Cancer Inst..

[227] Kouros-Mehr H., Werb Z. (2006). Candidate regulators of mammary branching morphogenesis identified by genome-wide transcript analysis. Dev. Dyn..

[228] Carroll T.J., Park J.S., Hayashi S., Majumdar A., McMahon A.P. (2005). Wnt9b plays a central role in the regulation of mesenchymal to epithelial transitions underlying organogenesis of the mammalian urogenital system. Dev. Cell..

[229] Ikenouchi J., Matsuda M., Furuse M., Tsukita S. (2003). Regulation of tight junctions during the epithelium-mesenchyme transition: Direct repression of the gene expression of claudins/occludin by Snail. J. Cell Sci..

[230] De Boer T.P., van Veen T.A., Bierhuizen M.F., Kok B., Rook M.B., Boonen K.J., Vos M.A., Doevendans P.A., de Bakker J.M., van der Heyden M.A. (2007). Connexin43 repression following epithelium-to-mesenchyme transition in embryonal carcinoma cells requires Snail1 transcription factor. Differentiation.

[231] Savanger P., Yamada K.M., Thiery J.P. (1997). The Zinc-Finger Protein Slug Causes Desmosome Dissociation, an Initial and Necessary Step for Growth Factor–induced Epithelial–Mesenchymal Transition. J. Cell Biol..

[232] Ciruna B., Rossant J. (2001). FGF signaling regulates mesoderm cell fate specification and morphogenetic movement at the primitive streak. Dev. Cell..

[233] Yook J.I., Li X.Y., Ota I., Hu C., Kim H.S., Kim N.H., Cha S.Y., Ryu J.K., Choi Y.J., Kim J. (2006). A Wnt-Axin2-GSK3β cascade regulates Snail1 activity in breast cancer cells. Nat. Cell Biol..

[234] Yu M., Smolen G.A., Zhang J., Wittner B., Schott B.J., Brachtel E., Ramaswamy S., Maheswaran S., Haber D.A. (2009). A developmentally regulated inducer of EMT, LBX1, contributes to breast cancer progression. Genes Dev..

[235] Peinado H., Quintanilla M., Cano A. (2003). Transforming growth factor β-1 induces snail transcription factor in epithelial cell lines: Mechanisms for epithelial mesenchymal transitions. J. Biol. Chem..

[236] Laffin B., Wellberg E., Kwak H.I., Burghardt R.C., Metz R.P., Gustafson T., Schedin P., Porter W.W. (2008). Loss of singleminded-2s in the mouse mammary gland induces an epithelial-mesenchymal transition associated with up-regulation of slug and matrix metalloprotease 2. Mol. Cell Biol..

[237] Cannito S., Novo E., Compagnone A., Valfrè di Bonzo L., Busletta C., Zamara E., Paternostro C., Povero D., Bandino A., Bozzo F. (2008). Redox mechanisms switch on hypoxia-dependent epithelial-mesenchymal transition in cancer cells. Carcinogenesis.

[238] Yang J., Mani S.A., Donaher J.L., Ramaswamy S., Itzykson R.A., Come C., Savagner P., Gitelman I., Richardson A., Weinberg R.A. (2004). Twist, a master regulator of morphogenesis, plays an essential role in tumor metastasis. Cell.

[239] Satoh K., Hamada S., Kimura K., Kanno A., Hirota M., Umino J., Fujibuchi W., Masamune A., Tanaka N., Miura K. (2008). Up-regulation of MSX2 enhances the malignant phenotype and is associated with twist 1 expression in human pancreatic cancer cells. Am. J. Pathol..

[240] Yang M.H., Wu K.J. (2008). TWIST activation by hypoxia inducible factor-1 (HIF-1): Implications in metastasis and development. Cell Cycle.

[241] Coletta R.D., McCoy E.L., Burns V., Kawakami K., McManaman J.L., Wysolmerski J.J., Ford H.L. (2010). Characterization of the Six1 homeobox gene in normal mammary gland morphogenesis. BMC Dev. Biol..

[242] Moffett P., Reece M., Pelletier J. (1997). The murine Sim-2 gene product inhibits transcription by active repression and functional interference. Mol. Cell Biol..

[243] Micalizzi D.S., Farabaugh S.M., Ford H.L. (2010). Epithelial-mesenchymal transition in cancer: Parallels between normal development and tumor progression. J. Mammary Gland Biol. Neoplasia.

[244] LaMarca H.L., Visbal A.P., Creighton C.J., Liu H., Zhang Y., Behbod F., Rosen J.M. (2010). CCAAT/enhancer binding protein β regulates stem cell activity and specifies luminal cell fate in the mammary gland. Stem. Cells.

[245] Siegel P.M., Muller W.J. (2010). Transcription factor regulatory networks in mammary epithelial development and tumorigenesis. Oncogene.

[246] Page-McCaw A., Ewald A.J., Werb Z. (2007). Matrix metalloproteinases and the regulation of tissue remodelling. Nat. Rev. Mol. Cell Biol..

[247] Khokha R., Werb Z. (2011). Mammary gland reprogramming: Metalloproteinases couple form with function. Cold Spring Harb. Perspect. Biol..

[248] Mori H., Lo A.T., Inman J.L., Alcaraz J., Ghajar C.M., Mott J.D., Nelson C.M., Chen C.S., Zhang H., Bascom J.L. (2013). Transmembrane/cytoplasmic, rather than catalytic, domains of Mmp14 signal to MAPK activation and mammary branching morphogenesis via binding to integrin β1. Development.

[249] Varner V.D., Voronov D.A., Taber L.A. (2010). Mechanics of head fold formation: Investigating tissue-level forces during early development. Development.

[250] Wiseman B.S., Sternlicht M.D., Lund L.R., Alexander C.M., Mott J., Bissell M.J., Soloway P., Itohara S., Werb Z. (2003). Site-specific inductive and inhibitory activities of MMP-2 and MMP-3 orchestrate mammary gland branching morphogenesis. J. Cell Biol..

[251] Szabova L., Yamada S.S., Birkedal-Hansen H., Holmbeck K. (2005). Expression pattern of four membrane-type matrix metalloproteinases in the normal and diseased mouse mammary gland. J. Cell Physiol..

[252] Schenk S., Hintermann E., Bilban M., Koshikawa N., Hojilla C., Khokha R., Quaranta V. (2003). Binding to EGF receptor of a laminin-5 EGF-like fragment liberated during MMP-dependent mammary gland involution. J. Cell Biol..

[253] Rebustini I.T., Myers C., Lassiter K.S., Surmak A., Szabova L., Holmbeck K., Pedchenko V., Hudson B.G., Hoffman M.P. (2009). MT2-MMP-dependent release of collagen IV NC1 domains regulates submandibular gland branching morphogenesis. Dev. Cell.

[254] Giannelli G., Falk-Marzillier J., Schiraldi O., Stetler-Stevenson W.G., Quaranta V. (1997). Induction of cell migration by matrix metalloprotease-2 cleavage of laminin-5. Science.

[255] Ucar A., Vafaizadeh V., Jarry H., Fiedler J., Klemmt P.A., Thum T., Groner B., Chowdhury K. (2010). miR-212 and miR-132 are required for epithelial stromal interactions necessary for mouse mammary gland development. Nat. Genet..

[256] Gomes A.M., Bhat R., Correia A.L., Mott J.D., Ilan N., Vlodavsky I., Pavão M.S.G., Bissell M. (2015). Mammary branching morphogenesis requires reciprocal signaling by heparanase and MMP-14. J. Cell. Biochem..

[257] Fernandez-Valdivia R., Mukherjee A., Ying Y., Li J., Paquet M., DeMayo F.J., Lydon J.P. (2009). The RANKL signaling axis is sufficient to elicit ductal side-branching and alveologenesis in the mammary gland of the virgin mouse. Dev. Biol..

[258] Mulac-Jericevic B., Lydon J.P., DeMayo F.J., Conneely O.M. (2003). Defective mammary gland morphogenesis in mice lacking the progesterone receptor B isoform. Proc. Natl. Acad. Sci. USA.

[259] Nelson C.M., Vanduijn M.M., Inman J.L., Fletcher D.A., Bissell M.J. (2006). Tissue geometry determines sites of mammary branching morphogenesis in organotypic cultures. Science.

[260] Taipale J., Miyazono K., Heldin C.H., Keski-Oja J. (1994). Latent transforming growth factor-β 1 associates to fibroblast extracellular matrix via latent TGF-β binding protein. J. Cell Biol..

[261] Robinson S.D., Silberstein G.B., Roberts A.B., Flanders K.C., Daniel C.W. (1991). Regulated expression and growth inhibitory effects of transforming growth factor-β isoforms in mouse mammary gland development. Development.

[262] Silberstein G.B., Daniel C.W. (1987). Reversible inhibition of mammary gland growth by transforming growth factor-β. Science.

[263] Munger J.S., Huang X., Kawakatsu H., Griffiths M.J., Dalton S.L., Wu J., Pittet J.F., Kaminski N., Garat C., Matthay M.A. (1999). The integrin αvβ6 binds and activates latent TGF β1: A mechanism for regulating pulmonary inflammation and fibrosis. Cell.

[264] Yu Q., Stamenkovic I. (2000). Cell surface-localized matrix metalloproteinase-9 proteolytically activates TGF-β and promotes tumor invasion and angiogenesis. Genes Dev..

[265] Wipff P.J., Rifkin D.B., Meister J.J., Hinz B. (2007). Myofibroblast contraction activates latent TGF-β1 from the extracellular matrix. J. Cell Biol..

[266] Massague J., Seoane J., Wotton D. (2005). Smad transcription factors. Genes Dev..

[267] Lee W.C., Davies J.A. (2007). Epithelial branching: The power of self-loathing. Bioessays.

[268] Pierce D.F., Johnson M.D., Matsui Y., Robinson S.D., Gold L.I., Purchio A.F., Daniel C.W., Hogan B.L., Moses H.L. (1993). Inhibition of mammary duct development but not alveolar outgrowth during pregnancy in transgenic mice expressing active TGF-β 1. Genes Dev..

[269] Silberstein G.B., Strickland P., Coleman S., Daniel C.W. (1990). Epithelium-dependent extracellular matrix synthesis in transforming growth factor-β 1-growth-inhibited mouse mammary gland. J. Cell Biol..

[270] Soriano J.V., Pepper M.S., Orci L., Montesano R. (1998). Roles of hepatocyte growth factor/scatter factor and transforming growth factor-β1 in mammary gland ductal morphogenesis. J. Mammary Gland Biol. Neoplasia.

[271] Sawhney R.K., Howard J. (2002). Slow local movements of collagen fibers by fibroblasts drive the rapid global self-organization of collagen gels. J. Cell Biol..

[272] Ballard M.S., Zhu A., Iwai N., Stensrud M., Mapps A., Postiglione M.P., Knoblich J.A., Hinck L. (2015). Mammary stem cell self-renewal is regulated by Slit2/Robo1 signaling through SNAI1 and mINSC. Cell Rep..

[273] Morris J.S., Stein T., Pringle M.A., Davies C.R., Weber-Hall S., Ferrier R.K., Bell A.K., Heath V.J., Gusterson B.A. (2006). Involvement of axonal guidance proteins and their signaling partners in the developing mouse mammary gland. J. Cell Physiol..

[274] Strickland P., Shin G.C., Plump A., Tessier-Lavigne M., Hinck L. (2006). Slit2 and netrin 1 act synergistically as adhesive cues to generate tubular bi-layers during ductal morphogenesis. Development.

[275] Chaffer C.L., Thompson E.W., Williams E.D. (2007). Mesenchymal to epithelial transition in development and disease. Cells Tissues Organs.

[276] Chrzanowska-Wodnicka M., Burridge K. (1996). Rho-stimulated contractility drives the formation of stress fibers and focal adhesions. J. Cell Biol..

[277] Landsverk M.L., Epstein H.F. (2005). Genetic analysis of myosin II assembly and organization in model organisms. Cell. Mol. Life Sci..

[278] Paszek M.J., Zahir N., Johnson K.R., Lakins J.N., Rozenberg G.I., Gefen A., Reinhart-King C.A., Margulies S.S., Dembo M., Boettiger D. (2005). Tensional homeostasis and the malignant phenotype. Cancer Cell..

[279] Tang B., de Castro K., Barnes H.E., Parks W.T., Stewart L., Böttinger E.P., Danielpour D., Wakefield L.M. (1999). Loss of responsiveness to transforming growth factor β induces malignant transformation of nontumorigenic rat prostate epithelial cells. Cancer Res..

[280] Wozniak M.A., Desai R., Solski P.A., Der C.J., Keely P.J. (2003). ROCK-generated contractility regulates breast epithelial cell differentiation in response to the physical properties of a three-dimensional collagen matrix. J. Cell Biol..

[281] Nagy T., Wie H., Shen T.L., Peng X., Liang C.C., Gan B., Guan J.L. (2007). Mammary epithelial-specific deletion of the focal adhesion kinase gene leads to severe lobulo-alveolar hypoplasia and secretory immaturity of the murine mammary gland. J. Biol. Chem..

[282] Van Miltenburg M.H., Lalai R., de Bont H., van Waaij E., Beggs H., Danen E.H., van de Water B. (2009). Complete focal adhesion kinase deficiency in the mammary gland causes ductal dilation and aberrant branching morphogenesis through defects in Rho kinase-dependent cell contractility. FASEB J..

[283] Kalluri R. (2009). EMT: When epithelial cells decide to become mesenchymal-like cells. J. Clin. Investig..

[284] Thiery J.P., Acloque H., Huang R.Y., Nieto M.A. (2009). Epithelial-mesenchymal transitions in development and disease. Cell.

[285] Revenu C., Gilmour D. (2009). EMT 2.0: Shaping epithelia through collective migration. Curr. Opin. Genet. Dev..

[286] Brahmbhatt A.A., Klemke R.L. (2003). ERK and RhoA differentially regulate pseudopodia growth and retraction during chemotaxis. J. Biol. Chem..

[287] Webb D.J., Parsons J.T., Horwitz A.F. (2002). Adhesion assembly, disassembly and turnover in migrating cells—Over and over and over again. Nat. Cell Biol..

[288] Ewald A.J., Brenot A., Duong M., Chan B.S., Werb Z. (2008). Collective epithelial migration and cell rearrangements drive mammary branching morphogenesis. Dev. Cell..

[289] Lu P., Werb Z. (2008). Patterning mechanisms of branched organs. Science.

[290] Nishimura T., Takeichi M. (2009). Remodeling of the adherens junctions during morphogenesis. Curr. Top Dev. Biol..

[291] Schedin P., Keely P.J. (2011). Mammary gland ECMremodeling, stiffness, and mechanosignaling in normal development and tumor progression. Cold Spring Harb. Perspect. Biol..

[292] Ramakrishnan R., Khan S.A., Badve S. (2002). Morphological changes in breast tissue with mestrual cycle. Mod. Pathol..

[293] Hartmann P.E. (1991). The breast and breast-feeding. Scientific Foundations of Obstetrics and Gynaecology.

[294] Moffatt D.F., Going J.J. (1996). Three dimensional anatomy of complete duct systems in the human breast: Pathological and developmental implications. J. Clin. Pathol..

[295] Love S.M., Barsky S.H. (2004). Anatomy of the nipple and breast ducts revisited. Cancer.

[296] Bannister L.H., Berry M.M., Collins P., Dyson M., Dussek J.E. (1995). Gray’s Anatomy.

[297] Jamal N., Ng K.H., McLean D., Looi L.M., Moosa F. (2004). Mammographic breast glandularity in malaysian women: Data derived from radiography. Am. J. Roentgenol..

[298] Cunningham L. (1977). The anatomy of the arteries and veins of the breast. J. Surg. Oncol..

[299] Schlenz I., Kuzbari R., Gruber H., Holle J. (2000). The sensitivity of the nipple-areola complex: An anatomic study. Plast. Reconstr. Surg..

[300] Das R., Vonderhaar B.K. (1997). Prolactin as a mitogen in mammary cells. J. Mammary Gland Biol. Neoplasia.

[301] Haslam S.Z. (1989). The ontogeny of mouse mammary gland responsiveness to ovarian steroid hormones. Endocrinology.

[302] Lydon J.P., DeMayo F.J., Funk C.R., Mani S.K., Hughes A.R., Montgomery C.A., Shyamala G., Conneely O.M., O’Malley B.W. (1995). Mice lacking progesterone receptor exhibit pleiotropic reproductive abnormalities. Genes Dev..

[303] Oakes S.R., Robertson F.G., Kench J.G., Gardiner-Garden M., Wand M.P., Green J.E., Ormandy C.J. (2007). Loss of mammary epithelial prolactin receptor delays tumor formation by reducing cell proliferation in low-grade preinvasive lesions. Oncogene.

[304] Oakes S.R., Rogers R.L., Naylor M.J., Ormandy C.J. (2008). Prolactin regulation of mammary gland development. J. Mammary Gland Biol. Neoplasia.

[305] Ormandy C.J., Binart N., Kelly P.A. (1997). Mammary gland development in prolactin receptor knockout mice. J. Mammary Gland Biol. Neoplasia.

[306] Russo J., Moral R., Balogh G.A., Mailo D., Russo I.H. (2005). The protective role of pregnancy in breast cancer. Breast Cancer Res..

[307] Blackman B., Russell T., Nordeenm S.K., Medina D., Neville M.C. (2005). Claudin 7 expression and localization in the normal murine mammary gland and murine mammary tumors. Breast Cancer Res..

[308] Rudolph M.C., McManaman J.L., Hunter L., Phang T., Neville M.C. (2003). Functional development of the mammary gland: Use of expression profiling and trajectory clustering to reveal changes in gene expression during pregnancy, lactation, and involution. J. Mammary Gland Biol. Neoplasia.

[309] Hennighausen L. (2001). The genetics and pathology of mouse mammary cancer. Semin. Cancer Biol..

[310] Neville M.C., Medina C., Monks J., Hovey R.C. (1998). Editorial Commentary: The mammary fat pad. J. Mammary Gland Biol. Neoplasia.

[311] Traurig H.H. (1967). A radioautographic study of cell proliferation in the mammary gland of the pregnant mouse. Anat. Rec..

[312] Thoresen M., Wesche J. (1988). Doppler measurements of changes in human mammary and uterine blood flow during pregnancy and lactation. Obstet. Gynecol. Scand.

[313] Hennighausen L., Robinson G.W. (2005). Information networks in the mammary gland. Nat. Rev. Mol. Cell Biol..

[314] D’Cruz C.M., Moody S.E., Master S.R., Hartman J.L., Keiper E.A., Imielinski M.B., Cox J.D., Wang J.Y., Ha S.I., Keister B.A. (2002). Persistent parity-induced changes in growth factors, TGF-β3, and differentiation in the rodent mammary gland. Mol. Endocrinol..

[315] Djonov V., Andres A.C., Ziemiecki A. (2001). Vascular remodelling during the normal and malignant life cycle of the mammary gland. Microsc. Res. Tech..

[316] McCready J., Arendt L.M., Rudnick J.A., Kuperwasser C. (2010). The contribution of dynamic stromal remodeling during mammary development to breast carcinogenesis. Breast Cancer Res..

[317] Milanese T.R., Hartmann L.C., Sellers T.A., Frost M.H., Vierkant R.A., Maloney S.D., Shane Pankratz V., Degnim A.C., Vachon C.M., Reynolds C.A. (2006). Age-related lobular involution and risk of breast cancer. J. Natl. Cancer Inst..

[318] Radisky D.C. (2009). Defining a role for the homeoprotein Six1 in EMT and mammary tumorigenesis. J. Clin. Investig..

[319] Brisken C., Ayyannan A., Nguyen C., Heineman A., Reinhardt F., Tan J., Dey S.K., Dotto G.P., Weinberg R.A. (2002). IGF-2 is a mediator of prolactin-induced morphogenesis in the breast. Dev. Cell..

[320] Haslam S.Z., Woodward T.L. (2003). Host microenvironment in breast cancer development: Epithelial-cell-stromal-cell interactions and steroid hormone action in normal and cancerous mammary gland. Breast Cancer Res..

[321] Streuli C.H., Akhtar N. (2009). Signal co-operation between integrins and other receptor systems. Biochem. J..

[322] Haslam S.Z., Levely M.L. (1985). Estrogen responsiveness of normal mouse mammary cells in primary cell culture: Association of mammary fibroblasts with estrogenic regulation of progesterone receptors. Endocrinology.

[323] Humphreys R.C., Lydon J., O’Malley B.W., Rosen J.M. (1997). Mammary gland development is mediated by both stromal and epithelial progesterone receptors. Mol. Endocrinol..

[324] Humphreys R.C., Lydon J., O’Malley B.W., Rosen J.M. (1997). Use of PRKO mice to study the role of progesterone in mammary gland development. J. Mammary Gland Biol. Neoplasia.

[325] Niswender G.D., Juengel J.L., Silva P.J., Rollyson M.K., McIntush E.W. (2000). Mechanisms controlling the function and life span of the corpus luteum. Physiol. Rev..

[326] Brisken C., Park S., Vass T., Lydon J.P., O’Malley B.W., Weinberg R.A. (1998). A paracrine role for the epithelial progesterone receptor in mammary gland development. Proc. Natl. Acad. Sci. USA.

[327] Humphreys R.C., Hennighausen L. (1999). Signal transducer and activator of transcription 5a influences mammary epithelial cell survival and tumorigenesis. Cell Growth Differ..

[328] Hiremath M., Lydon J.P., Cowin P. (2007). The pattern of β-catenin responsiveness within the mammary gland is regulated by progesterone receptor. Development.

[329] Teissedre B., Pinderhughes A., Incassati A., Hatsell S.J., Hiremath M., Cowin P. (2009). MMTV-Wnt1 and -ΔN89β-catenin induce canonical signaling in distinct progenitors and differentially activate Hedgehog signaling within mammary tumors. PLoS ONE.

[330] Brisken C., Heineman A., Chavarria T., Elenbaas B., Tan J., Dey S.K., McMahon J.A., McMahon A.P., Weinberg R.A. (2000). Essential function of Wnt-4 in mammary gland development downstream of progesterone signaling. Genes Dev..

[331] Brisken C., Kaur S., Chavarria T.E., Binart N., Sutherland R.L., Weinberg R.A., Kelly P.A., Ormandy C.J. (1999). Prolactin controls mammary gland development via direct and indirect mechanisms. Dev. Biol..

[332] Ruan W., Monaco M.E., Kleinberg D.L. (2005). Progesterone stimulates mammary gland ductal morphogenesis by synergizing with and enhancing insulin-like growth factor-I action. Endocrinology.

[333] Schramek D., Leibbrandt A., Sigl V., Kenner L., Pospisilik J.A., Lee H.J., Hanada R., Joshi P.A., Aliprantis A., Glimcher L. (2010). Osteoclast differentiation factor RANKL controls development of progestin-driven mammary cancer. Nature.

[334] Theill L.E., Boyle W.J., Penninger J.M. (2002). RANK-L and RANK: T cells, bone loss, and mammalian evolution. Annu. Rev. Immunol..

[335] Lamberti C., Lin K.M., Yamamoto Y., Verma U., Verma I.M., Byers S., Gaynor R.B. (2001). Regulation of β-catenin function by the IκB kinases. J. Biol. Chem..

[336] Gavin B.J., McMahon A.P. (1992). Differential regulation of the Wnt gene family during pregnancy and lactation suggests a role in postnatal development of the mammary gland. Mol. Cell Biol..

[337] Weber-Hall S.J., Phippard D.J., Niemeyer C.C., Dale T.C. (1994). Developmental and hormonal regulation of Wnt gene expression in the mouse mammary gland. Differentiation.

[338] Kim Y.C., Clark R.J., Pelegri F., Alexander C.M. (2009). Wnt4 is not sufficient to induce lobuloalveolar mammary development. BMC Dev. Biol..

[339] Freeman M.E., Kanyicska B., Lerant A., Nagy G. (2000). 2000 Prolactin: Structure, function, and regulation of secretion. Physiol. Rev..

[340] Hennighausen L., Robinson G.W. (1998). Think globally, act locally: The making of a mouse mammary gland. Genes Dev..

[341] Horseman N.D., Zhao W., Montecino-Rodriguez E., Tanaka M., Nakashima K., Engle S.J., Smith F., Markoff E., Dorshkind K. (1997). Defective mammopoiesis, but normal hematopoiesis, in mice with a targeted disruption of the prolactin gene. EMBO J..

[342] Liu X., Robinson G.W., Wagner K.U., Garrett L., Wynshaw-Boris A., Hennighausen L. (1997). Stat5a is mandatory for adult mammary gland development and lactogenesis. Genes Dev..

[343] Miyoshi K., Cui Y., Riedlinger G., Robinson P., Lehoczky J., Zon L., Oka T., Dewar K., Hennighausen L. (2001). Structure of the mouse Stat 3/5 locus: Evolution from Drosophila to zebrafish to mouse. Genomics.

[344] Udy G.B., Towers R.P., Snell R.G., Wilkins R.J., Park S.H., Ram P.A., Waxman D.J., Davey H.W. (1997). Requirement of STAT5b for sexual dimorphism of body growth rates and liver gene expression. Proc. Natl. Acad. Sci. USA.

[345] Zhou J., Chehab R., Tkalcevic J., Naylor M.J., Harris J., Wilson T.J., Tsao S., Tellis I., Zavarsek S., Xu D. (2005). Elf5 is essential for early embryogenesis and mammary gland development during pregnancy and lactation. EMBO J..

[346] Harris J., Stanford P.M., Sutherland K., Oakes S.R., Naylor M.J., Robertson F.G., Blazek K.D., Kazlauskas M., Hilton H.N., Wittlin S. (2006). Socs2 and elf5 mediate prolactin-induced mammary gland development. Mol. Endocrinol..

[347] Kubo M., Hanada T., Yoshimura A. (2003). Suppressors of cytokine signaling and immunity. Nat. Immunol..

[348] Lindeman G.J., Wittlin S., Lada H., Naylor M.J., Santamaria M., Zhang J.G., Starr R., Hilton D.J., Alexander W.S., Ormandy C.J. (2001). SOCS1 deficiency results in accelerated mammary gland development and rescues lactation in prolactin receptor-deficient mice. Genes Dev..

[349] Park D.S., Lee H., Frank P.G., Razani B., Nguyen A.V., Parlow A.F., Russell R.G., Hulit J., Pestell R.G., Lisanti M.P. (2002). Caveolin-1-deficient mice show accelerated mammary gland development during pregnancy, premature lactation, and hyperactivation of the Jak-2/STAT5a signaling cascade. Mol. Biol. Cell.

[350] Srivastava S., Matsuda M., Hou Z., Bailey J.P., Kitazawa R., Herbst M.P., Horseman N.D. (2003). Receptor activator of NF-κB ligand induction via Jak2 and Stat5a in mammary epithelial cells. J. Biol. Chem..

[351] Fantl V., Stamp G., Andrews A., Rosewell I., Dickson C. (1995). Mice lacking cyclin D1 are small and show defects in eye and mammary gland development. Genes Dev..

[352] Sicinski P., Donaher J.L., Parker S.B., Li T., Fazeli A., Gardner H., Haslam S.Z., Bronson R.T., Elledge S.J., Weinberg R.A. (1995). Cyclin D1 provides a link between development and oncogenesis in the retina and breast. Cell.

[353] Li S., Rosen J.M. (1994). Distal regulatory elements required for rat whey acidic protein gene expression in transgenic mice. J. Biol. Chem..

[354] Pittius C.W., Sankaran L., Topper Y.J., Hennighausen L. (1988). Comparison of the regulation of the whey acidic protein gene with that of a hybrid gene containing the whey acidic protein gene promoter in transgenic mice. Mol. Endocrinol..

[355] Walton K.D., Wagner K.U., Rucker E.B., Shillingford J.M., Miyoshi K., Hennighausen L. (2001). Conditional deletion of the bcl-x gene from mouse mammary epithelium results in accelerated apoptosis during involution but does not compromise cell function during lactation. Mech. Dev..

[356] Khaled W.T., Read E.K., Nicholson S.E., Baxter F.O., Brennan A.J., Came P.J., Sprigg N., McKenzie A.N., Watson C.J. (2007). The IL-4/IL-13/Stat6 signalling pathway promotes luminal mammary epithelial cell development. Development.

[357] Barcellos-Hoff M.H., Aggeler J., Ram T.G., Bissell M.J. (1989). Functional differentiation and alveolar morphogenesis of primary mammary cultures on reconstituted basement membrane. Development.

[358] Katz E., Streuli C.H. (2007). The extracellular matrix as an adhesion checkpoint for mammary epithelial function. Int. J. Biochem. Cell Biol..

[359] Streuli C.H., Bissell M.J. (1991). Expression of extracellular matrix components is regulated by substratum. J. Cell Biol..

[360] Streuli C.H., Edwards G.M., Delcommenne M., Whitelaw C.B., Burdon T.G., Schindler C., Watson C.J. (1995). Stat5 as a target for regulation by extracellular matrix. J. Biol. Chem..

[361] Choi Y.S., Chakrabarti R., Escamilla-Hernandez R., Sinha S. (2009). Elf5 conditional knockout mice reveal its role as a master regulator in mammary alveolar development: Failure of Stat5 activation and functional differentiation in the absence of Elf5. Dev. Biol..

[362] Lapinskas E.J., Palmer J., Ricardo S., Hertzog P.J., Hammacher A., Pritchard M.A. (2004). A major site of expression of the ets transcription factor Elf5 is epithelia of exocrine glands. Histochem. Cell Biol..

[363] Gilbert S.F. (2006). Developmental Biology.

[364] Antonova L., Aronson K., Mueller C.R. (2011). Stress and breast cancer: From epidemiology to molecular biology. Breast Cancer Res..

[365] Homo-Delarche F., Fitzpatrick F., Christeff N., Nunez E.A., Bach J.F., Dardenne M. (1991). Sex steroids, glucocorticoids, stress and autoimmunity. J. Steroid Biochem. Mol. Biol..

[366] VanItallie T. (2002). Stress: A risk factor for serious illness. Metabolism.

[367] Majumder P., Joshi J., Banerjee M. (1983). Correlation between nuclear glucocorticoid receptor levels and casein gene expression in murine mammary gland in vitro. J. Biol. Chem..

[368] Reichardt H., Horsch K., Grone H., Kolbus A., Beug H., Hynes N., Schutz G. (2001). Mammary gland development and lactation are controlled by diff erent glucocorticoid receptor activities. Eur. J. Endocrinol..

[369] Wintermantel T., Bock D., Fleig V., Greiner E., Schutz G. (2005). The epithelial glucocorticoid receptor is required for the normal timing of cell proliferation during mammary lobuloalveolar development but is dispensable for milk production. Mol. Endocrinol..

[370] Jones F.E., Welte T., Fu X.Y., Stern D.F. (1999). ErbB4 signaling in the mammary gland is required for lobuloalveolar development and Stat5 activation during lactation. J. Cell Biol..

[371] Muraoka-Cook R.S., Sandahl M., Hunter D., Miraglia L., Earp H.S. (2008). Prolactin and ErbB4/HER4 signaling interact via Janus kinase 2 to induce mammary epithelial cell gene expression differentiation. Mol. Endocrinol..

[372] Ball E.M., Risbridger G.P. (2001). Activins as regulators of branching morphogenesis. Dev. Biol..

[373] Bloise E., Cassali G.D., Ferreira M.C., Ciarmela P., Petraglia F., Reis F.M. (2010). Activin-related proteins in bovine mammary gland: Localization and differential expression during gestational development and differentiation. J. Dairy Sci..

[374] Muttukrishna S., Hyett J., Paine M., Moodley J., Groome N., Rodeck C. (2006). Uterine vein and maternal urinary levels of activin A and inhibin A in pre-eclampsia patients. Clin. Endocrinol..

[375] Luisi S., Calonaci G., Florio P., Lombardi I., De Felice C., Bagnoli F., Petraglia F. (2002). Identification of activin A and follistatin in human milk. Growth Factors.

[376] Bussmann U.A., Lanuza G.M., Bussmann L.E. (2004). Activin and follistatin in rat mammary gland. Mol. Cell Endocrinol..

[377] Gorska A.E., Jensen R.A., Shyr Y., Aakre M.E., Bhowmick N.A., Moses H.L. (2003). Transgenic mice expressing a dominant-negative mutant type II transforming growth factor-β receptor exhibit impaired mammary development and enhanced mammary tumor formation. Am. J. Pathol..

[378] Adair-Kirk T.L., Senior R.M. (2008). Fragments of extracellular matrix as mediators of inflammation. Int. J. Biochem. Cell Biol..

[379] Huang W., Chiquet-Ehrismann R., Moyano J.V., Garcia-Pardo A., Orend G. (2001). Interference of tenascin-C with syndecan-4 binding to fibronectin blocks cell adhesion and stimulates tumor cell proliferation. Cancer Res..

[380] Li N., Zhang Y., Naylor M.J., Schatzmann F., Maurer F., Wintermantel T., Schuetz G., Mueller U., Streuli C.H., Hynes N.E. (2005). β1 integrins regulate mammary gland proliferation and maintain the integrity of mammary alveoli. EMBO J..

[381] Akhtar N., Marlow R., Lambert E., Schatzmann F., Lowe E.T., Cheung J., Katz E., Li W., Wu C., Dedhar S. (2009). Molecular dissection of integrin signalling proteins in the control of mammary epithelial development and differentiation. Development.

[382] Akhtar N., Streuli C.H. (2006). Rac1 links integrin-mediated adhesion to the control of lactational differentiation in mammary epithelia. J. Cell Biol..

[383] Schedin P., O’Brien J., Rudolph M., Stein T., Borges V. (2007). Microenvironment of the involuting mamJmary gland mediates mammary cancer progression. J. Mammary Gland Biol. Neoplasia.

[384] O’Brien J., Lyons T., Monks J., Lucia M.S., Wilson R.S., Hines L., Man Y.G., Borges V., Schedin P. (2010). Alternatively activated macrophages and collagen remodeling characterize the postpartum involuting mammary gland across species. Am. J. Pathol..

[385] Humphreys R.C., Bierie B., Zhao L., Raz R., Levy D., Hennighausen L. (2002). Deletion of Stat3 blocks mammary gland involution and extends functional competence of the secretory epithelium in the absence of lactogenic stimuli. Endocrinology.

[386] Mailleux A.A., Overholtzer M., Schmelzle T., Bouillet P., Strasser A., Brugge J.S. (2007). BIM regulates apoptosis during mammary ductal morphogenesis, and its absence reveals alternative cell death mechanisms. Dev. Cell.

[387] Wang N., Kudryavtseva E., Chen I.L., McCormick J., Sugihara T.M., Ruiz R., Andersen B. (2004). Expression of an engrailed-LMO4 fusion protein in mammary epithelial cells inhibits mammary gland development in mice. Oncogene.

[388] Whyte J., Bergin O., Bianchi A., McNally S., Martin F. (2009). Key signalling nodes in mammary gland development and cancer. Mitogen-activated protein kinase signalling in experimental models of breast cancer progression and in mammary gland development. Breast Cancer Res..

[389] Pearson J.F., Hughes S., Chambers K., Lang S.H. (2009). Polarized fluid movement and not cell death, creates luminal spaces in adult prostate epithelium. Cell Death Differ..

[390] Weir M.L., Oppizzi M.L., Henry M.D., Onishi A., Campbell K.P., Bissell M.J., Muschler J.L. (2006). Dystroglycan loss disrupts polarity and β-casein induction in mammary epithelial cells by perturbing laminin anchoring. J. Cell Sci..

[391] Alcaraz J., Xu R., Mori H., Nelson C.M., Mroue R., Spencer V.A., Brownfield D., Radisky D.C., Bustamante C., Bissell M.J. (2008). Laminin and biomimetic extracellular elasticity enhance functional differentiation in mammary epithelia. EMBO J..

[392] Prajapati R.T., Chavally-Mis B., Herbage D., Eastwood M., Brown R.A. (2000). Mechanical loading regulates protease production by fibroblasts in three-dimensional collagen substrates. Wound Repair Regen..

[393] Ruddy J.M., Jones J.A., Stroud R.E., Mukherjee R., Spinale F.G., Ikonomidis J.S. (2009). Differential effects of mechanical and biological stimuli on matrix metalloproteinase promoter activation in the thoracic aorta. Circulation.

[394] McManaman J.L., Neville M.C. (2003). Mammary physiology and milk secretion. Adv. Drug Deliv. Rev..

[395] Monks J., Rosner D., Jon Geske F., Lehman L., Hanson L., Neville M.C., Fadok V.A. (2005). Epithelial cells as phagocytes: Apoptotic epithelial cells are engulfed by mammary alveolar epithelial cells and repress inflammatory mediator release. Cell Death Differ..

[396] Monks J., Smith-Steinhart C., Kruk E.R., Fadok V.A., Henson P.M. (2008). Epithelial cells remove apoptotic epithelial cells during post-lactation involution of the mouse mammary gland. Biol. Reprod..

[397] Lim C.L., Yu Zuan Or Y.Z., Ong Z., Chung H.H., Hayashi H. (2020). Estrogen exacerbates mammary involution through neutrophil-dependent and –independent mechanism. eLife.

[398] Chapman R.S., Lourenco P., Tonner E., Flint D., Selbert S., Takeda K., Akira S., Clarke A.R., Watson C.J. (2000). The role of Stat3 in apoptosis and mammary gland involution. Conditional deletion of Stat3. Adv. Exp. Med. Biol..

[399] Håkansson A., Andréasson J., Zhivotovsky B., Karpman D., Orrenius S., Svanborg C. (1999). Multimeric α-lactalbumin from human milk induces apoptosis through a direct effect on cell nuclei. Exp Cell Res..

[400] Håkansson A., Zhivotovsky B., Orrenius S., Sabharwal H., Svanborg C. (1995). Apoptosis induced by a human milk protein. Proc. Natl. Acad. Sci. USA.

[401] Kritikou E.A., Sharkey A., Abell K., Came P.J., Anderson E., Clarkson R.W., Watson C.J. (2003). A dual, non-redundant, role for LIF as a regulator of development and STAT3-mediated cell death in mammary gland. Development.

[402] Schere-Levy C., Buggiano V., Quaglino A., Gattelli A., Cirio M.C., Piazzon I., Vanzulli S., Kordon E.C. (2003). Leukemia inhibitory factor induces apoptosis of the mammary epithelial cells and participates in mouse mammary gland involution. Exp. Cell Res..

[403] Chapman R.S., Lourenco P.C., Tonner E., Flint D.J., Selbert S., Takeda K., Akira S., Clarke A.R., Watson C.J. (1999). Suppression of epithelial apoptosis and delayed mammary gland involution in mice with a conditional knockout of Stat3. Genes Dev..

[404] Yang Y.A., Tang B., Robinson G., Hennighausen L., Brodie S.G., Deng C.X., Wakefield L.M. (2002). Smad3 in the mammary epithelium has a nonredundant role in the induction of apoptosis, but not in the regulation of proliferation or differentiation by transforming growth factor-β. Cell Growth Differ..

[405] Roberts A.W., Robb L., Rakar S., Hartley L., Cluse L., Nicola N.A., Metcalf D., Hilton D.J., Alexander W.S. (2001). Placental defects and embryonic lethality in mice lacking suppressor of cytokine signaling. Proc. Natl. Acad. Sci. USA.

[406] Zhao L., Hart S., Cheng J., Melenhorst J.J., Bierie B., Ernst M., Stewart C., Schaper F., Heinrich P.C., Ullrich A. (2004). Mammary gland remodeling depends on gp130 signaling through Stat3 and MAPK. J. Biol. Chem..

[407] Zhao L., Melenhorst J.J., Hennighausen L. (2002). Loss of interleukin 6 results in delayed mammary gland involution: A possible role for mitogen-activated protein kinase and not signal transducer and activator of transcription. Mol. Endocrinol..

[408] Takahashi Y., Carpino N., Cross J.C., Torres M., Parganas E., Ihle J.N. (2003). SOCS3: An essential regulator of LIF receptor signaling in trophoblast giant cell differentiation. EMBO J..

[409] Sun H., Miao Z., Zhang X., In Chan U., Su S.M., Guo S., Ho Wong C.K., Xiaoling X., Chu-Xia Deng X. (2018). Single-cell RNA-Seq reveals cell heterogeneity and hierarchy within mouse mammary epithelia. J. Biol. Chem..

[410] Chung C.Y., Ma Z., Dravis C., Preissl S., Poirion O., Luna G., Hou X., Giraddi R.R., Ren B., Wahl G.M. (2019). Single-Cell Chromatin Analysis of Mammary Gland Development Reveals Cell-State Transcriptional Regulators and Lineage Relationships. Cell Rep..

[411] Bach K., Pensa S., Grzelak M., Hadfield J., Adams D.J., Marioni J.C., Khaled W.T. (2017). Differentiation dynamics of mammary epithelial cells revealed by single-cell RNA sequencing. Nat. Commun..

[412] Twigger A.J., Khaled W.T. (2021). Mammary gland development from a single cell omics view. Semin. Cell Devel. Biol..

[413] Gudjonsson T., Villadsen R., Nielsen H.L., Rønnov-Jessen L., Bissell M.J., Petersen O.W. (2002). Isolation, immortalization, and characterization of a human breast epithelial cell line with stem cell properties. Genes Dev..

